# Genome-wide DNA methylation analysis of KRAS mutant cell lines

**DOI:** 10.1038/s41598-020-66797-x

**Published:** 2020-06-23

**Authors:** Ben Yi Tew, Joel K. Durand, Kirsten L. Bryant, Tikvah K. Hayes, Sen Peng, Nhan L. Tran, Gerald C. Gooden, David N. Buckley, Channing J. Der, Albert S. Baldwin, Bodour Salhia

**Affiliations:** 1 Department of Translational Genomics, University of Southern California, Los Angeles, CA 90033 USA; 2 Lineberger Comprehensive Cancer Center, University of North Carolina at Chapel Hill, Chapel Hill, NC 27599 USA; 3 Cancer and Cell Biology Division, Translational Genomics Research Institute, Phoenix, AZ 85004 USA; 4 Departments of Cancer Biology and Neurology, Mayo Clinic Arizona, Scottsdale, AZ 85259 USA

**Keywords:** Cancer epigenetics, Oncogenes

## Abstract

Oncogenic *RAS* mutations are associated with DNA methylation changes that alter gene expression to drive cancer. Recent studies suggest that DNA methylation changes may be stochastic in nature, while other groups propose distinct signaling pathways responsible for aberrant methylation. Better understanding of DNA methylation events associated with oncogenic KRAS expression could enhance therapeutic approaches. Here we analyzed the basal CpG methylation of 11 KRAS-mutant and dependent pancreatic cancer cell lines and observed strikingly similar methylation patterns. KRAS knockdown resulted in unique methylation changes with limited overlap between each cell line. In KRAS-mutant Pa16C pancreatic cancer cells, while KRAS knockdown resulted in over 8,000 differentially methylated (DM) CpGs, treatment with the ERK1/2-selective inhibitor SCH772984 showed less than 40 DM CpGs, suggesting that ERK is not a broadly active driver of KRAS-associated DNA methylation. KRAS G12V overexpression in an isogenic lung model reveals >50,600 DM CpGs compared to non-transformed controls. In lung and pancreatic cells, gene ontology analyses of DM promoters show an enrichment for genes involved in differentiation and development. Taken all together, KRAS-mediated DNA methylation are stochastic and independent of canonical downstream effector signaling. These epigenetically altered genes associated with KRAS expression could represent potential therapeutic targets in KRAS-driven cancer.

## Introduction

Activating KRAS mutations can be found in nearly 25 percent of all cancers^1^. Pancreatic and lung cancers, in particular, exhibit high rates of oncogenic KRAS mutation, at 95% and 30%, respectively^2^. In this respect, KRAS has been established as a crucial oncoprotein in the progression and maintenance of KRAS-mutant pancreatic and lung cancers^3–8^. The important role of oncogenic KRAS in cancer has been met with nearly four decades of effort to develop therapeutic strategies to target aberrant KRAS function for cancer treatment^9,10^. Recently, direct inhibitors of mutant KRAS have been developed^10,11^, and have entered clinical evaluation^12^. While the G12C mutation is prevalent in KRAS-mutant lung adenocarcinoma (~46%), this mutation is found in only 2% of PDAC^13^. Therefore, indirect approaches remain the best option for the majority of KRAS-mutant PDAC. Among indirect approaches, the inhibition of downstream effectors, the RAF-MEK-ERK MAPK cascade and the PI3K-AKT-mTOR pathways, remain the most promising direction^14–18^.

In addition to aberrant effector signaling, most cancer cells also undergo genome-scale epigenetic changes. The most widely studied biochemical modification governing epigenetics is DNA methylation of CpG dinucleotides^19^. DNA methylation in mammalian organisms occurs by the covalent addition of a methyl group to the C-5 position of cytosine base in a CpG sequence context. The human genome is CpG depleted, while nearly 70% of all CpGs are methylated, mostly in transposable elements and intergenic regions of the human genome. DNA methylation can impact proximal chromatin structure and regulate gene expression, playing critical roles in biological processes including embryonic development, X-chromosome inactivation, genomic imprinting, and chromosome stability^19^. Hence, determining the methylation status at a single base resolution in the genome is an important step in elucidating its role in regulating many cellular processes and its disruption in disease states. CpG methylation can be dynamically regulated and this process is reversible.

Global DNA hypomethylation and focal hypermethylation at CpG islands have become hallmarks of cancer^20–23^. Moreover, oncogenic KRAS expression has specifically been shown to induce aberrant DNA methylation, promoting hypomethylation across the genome while silencing key tumor suppressors through hypermethylation^24–27^. Gazin *et al*.^24^ reported an ordered pathway associated with RAS-induced epigenetic signaling. KRAS-associated differential DNA methylation could have a significant impact across the genome and lead to important oncogenic transcriptional changes. Discovering an essential and predictable epigenetic response to mutant KRAS expression either within one cancer type, across multiple cancer types, or specificity to a particular KRAS mutation (i.e. G12D), could reveal other potential anti-cancer targets. Interestingly, Xie *et al*.^28^. found that HRAS-transformed cells show methylation patterns diverging dramatically from reproducible methylation pattern of senescence. The authors suggest that cell transformation involves stochastic epigenetic patterns from which malignant cells may evolve. Ultimately, a better understanding of the DNA methylation events associated with oncogenic KRAS expression could enhance therapeutic approaches for KRAS-driven cancers and provide a platform for understanding the intrinsic heterogeneous nature of these cancers.

We have previously shown that mutant KRAS drives distinct molecular changes in pancreatic^29^ and lung^30^ cancer cells. However, it remains unclear whether these molecular changes are associated with epigenetic changes. Here we perform a genome-scale analysis using KRAS-mutant human pancreatic and lung cancer cell lines to investigate whether knock-down or overexpression of mutant KRAS as well as pharmacological inhibtion of ERK correlates with differential DNA methylation. We found that while KRAS-mediated DNA methylation changes were cell type specific, gene ontology analysis revealed that many of the genes were associated with development and differentiation. Furthermore, we found that ERK inhibition did not reverse the great majority of KRAS-mediated methylation changes, suggesting that ERK is not a main driver for KRAS-mediated DNA methylation changes.

## Results

### CpG methylation in a panel of 47 cell lines shows clustering of cell lines with similar tissue of origin independent of *KRAS* mutation status

**Figure 1:**
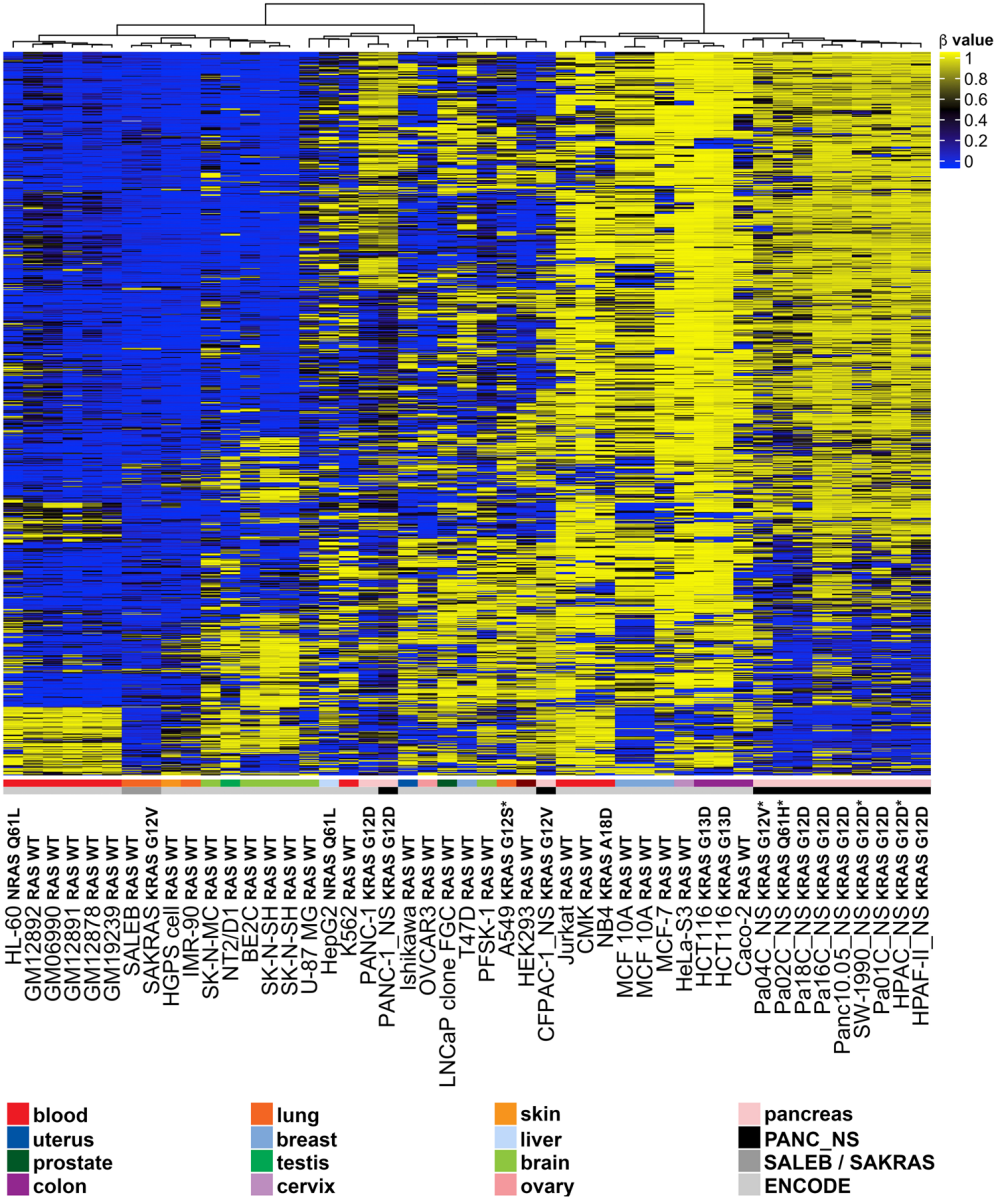
CpG methylation in a panel of 47 cell lines with varying KRAS status. Unsupervised hierarchical clustering analysis using the top 1000 most variable CpG probes across a panel of 47 cell lines is displayed above. Eleven human pancreatic cancer cell lines were transduced with non-silencing (NS) shRNA (black bar above). DNA methylation patterns in these pancreatic cells were compared to the DNA methylation in lung epithelial SALEB/SAKRAS cells and Infinium methylation data obtained from ENCODE (www.encodeproject.org). The β value for each probe is represented with a color scale as shown in the key. Values closer to 1 represent highly methylated CpGs, while values closer to zero represent least methylated CpGs.

Given the essential role of oncogenic KRAS in the great majority of pancreatic cancer^15,29^ (see cell line information, Supplementary Fig. S1), we investigated whether the presence of an activating *KRAS* mutation correlates with specific patterns of global DNA methylation. We first performed genome-wide DNA methylation profiling of 11 KRAS-dependent pancreatic cancer cell lines using the Infinium HumanMethylation450 BeadChip Array^31^. We also surveyed the CpG methylation patterns in low passage, immortalized lung epithelial cells transduced with KRAS G12V (SAKRAS cells) and non-transformed empty vector controls (SALEB cells). We compared the panel of 11 KRAS-mutant pancreatic cancer cell lines to DNA methylation data collected from SALEB and SAKRAS lung epithelial cells and published Infinium methylation data from ENCODE^32^ (Fig. 1). The published ENCODE data include three non-transformed human cell lines (HGPS and IMR-90 fibroblasts, and two different MCF 10 A breast epithelial cell lines) and 30 cell lines of varying cell types, genetic backgrounds, and tumorigenicity. As the pancreatic cancer cell lines were transduced with non-silencing (NS) shRNA, which could potentially affect the methylome of the transduced cells, we performed the same analysis while excluding these cells (Supplementary Fig. S2). After unsupervised hierachial clustering of the top 1,000 most variable CpG probes across all 47 cell lines, the pancreatic cancer cell lines formed a distinct cluster with the exception of CFPAC-1_NS and PANC-1_NS cells. These data suggest that the panel of KRAS-mutant pancreatic cancer cell lines contain similar overall basal DNA methylation patterns. Other KRAS mutant lines were clustered in the same branch of the dendrogram. However, in general, the cell lines formed clusters based on cell type with a few exceptions, and this was true regardless of the exclusion of the transduced pancreatic cancer cell lines. This suggests that even as KRAS may influence some key changes to the epigenome, DNA methylation patterns observed are more influenced by cell type.

### Unsupervised hierachical clustering shows cell line specific differential CpG methylation associated with *KRAS* suppression in pancreatic cancer cells

**Figure 2:**
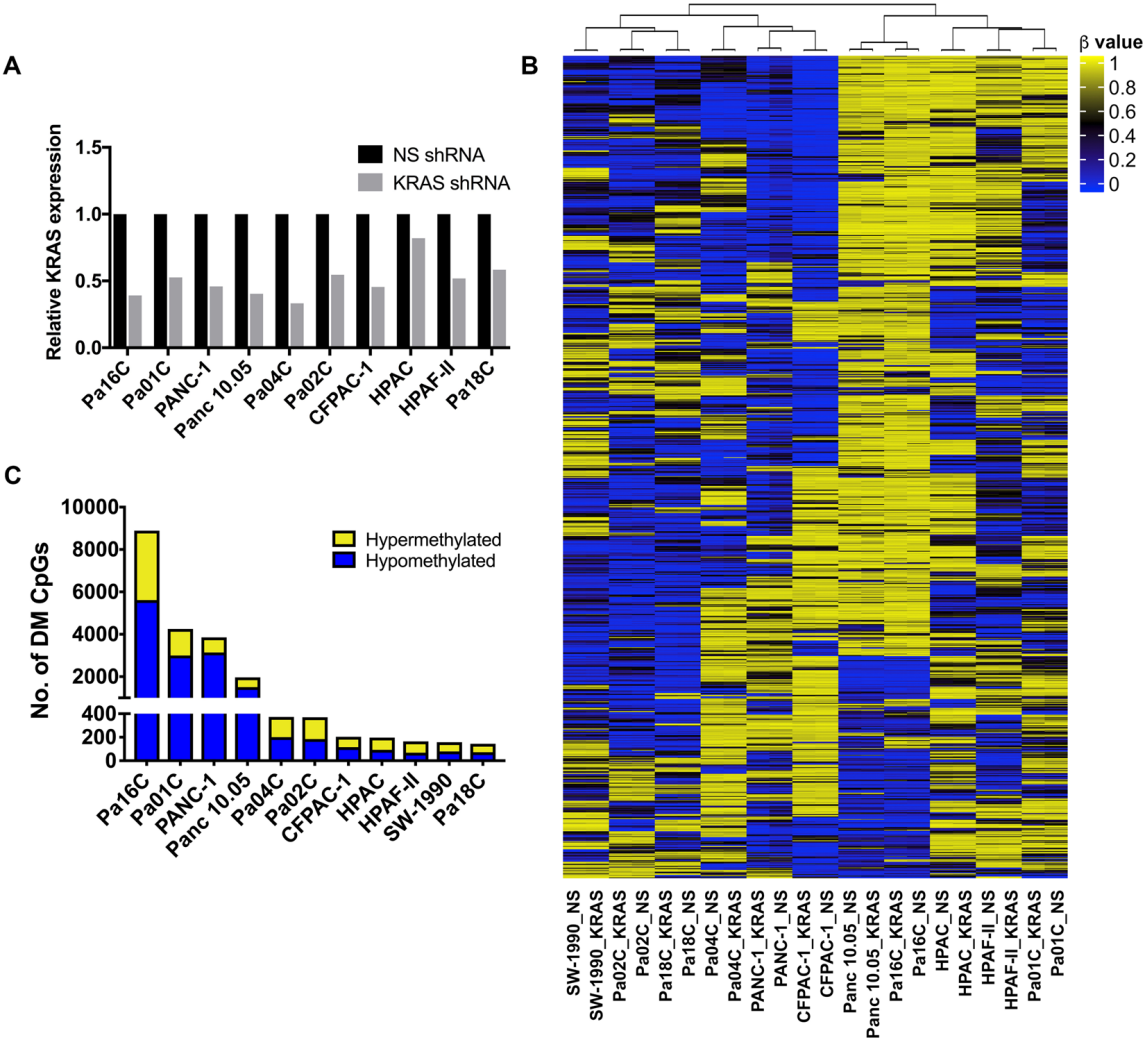
Effects of KRAS inhibition on DNA methylation. (**A**) KRAS mRNA levels from 10 pancreatic cancer cell lines transduced with KRAS shRNA compared to non-silencing (NS) controls as measured by RNA sequencing. RNA was not collected for SW-1990 cells due to insufficient material. **(B)** Unsupervised hierarchical clustering analysis was performed using the top 1000 most variable CpG probes across the panel of 11 pancreatic cell lines transduced with NS shRNA or KRAS shRNA. The β value for each probe is represented with a color scale as shown in the key. **(C)** Bar graph showing the number of differentially methylated (DM) CpGs with Δβ values ≥0.2 or ≤−0.2 in cell lines transduced with KRAS shRNA (hypermethylated CpGs represented in yellow and hypomethylated CpGs represented in blue).

We have previously shown that silencing KRAS caused distinct molecular changes in pancreatic cancer cell lines^29^. Silencing of KRAS may therefore also lead to differential DNA methylation. To test this, we performed RNA-seq and genome-wide DNA methylation analysis using Illumina’s Infinium arrays to determine the effect of silencing of *KRAS* in the 11 KRAS-mutant and -dependent pancreatic cancer cells. Briefly, cells were harvested for RNA and genomic DNA 4 to 7 days following infection with lentivirus shRNA targeting KRAS. Despite being KRAS-dependent, KRAS knockdown was not sufficient to cause dramatic cell death in pancreatic cell lines. This has been observed previously, and these cells lines were shown to be able to activate compensatory pathways in response to KRAS suppression^29^. Reduced KRAS mRNA levels were observed in KRAS-depleted cells relative to NS controls as determined by RNA sequencing (Fig. 2A). We then performed GSEA to compare the KRAS-depleted cells to the NS controls, and found a reduction in KRAS signaling (Supplementary Fig. S3), as evident from decreased enrichment in genes which are upregulated by KRAS (HALLMARK_KRAS_UP) and increase enrichment in genes downregulated by KRAS (HALLMARK_KRAS_DN). There was also a decrease in both PI3K/AKT and mTORC signaling, which are pathways downstream of KRAS.

Unsupervised hierarchical clustering of genome-wide DNA methylation data using the top 1000 most variable CpG probes revealed co-clustering of isogenic cell line pairs in that all 11 KRAS-depleted cell lines and their isogenic controls appear more similar to each other than any other cell line (Fig. 2B). There was also no clear separation based on specific KRAS mutations (G12D vs G12V). There were two distinct branches separating the 11 isogeneic pairs (Fig. 2B), representing a group with a lower degree of methylation than the other. To identify common methylation changes between the pancreatic cell lines, we performed heirachical clustering using the union of differentially methylated probes (Δβ values ≥0.2 or ≤−0.2) appearing in at least 3 out of the 11 cell line pairs (a total of 204 CpG probes) and observed co-clustering of Pa16C, Pa01C, and Panc 10.05 (Supplementary Fig. S4A). From this list of 204 CpG probes, which represents the most frequently differentially methylated (DM) probes, we compiled the top 10 DM hypo or hypermethylated CpGs per cell line into a list (Supplementary Fig. S4B).

Next we examined the number of DM probes per cell line as a measure of the extent of DNA methylation response due to KRAS inhibition. The DNA methylation profiles of Pa16C, Pa01C, PANC-1, and Panc 10.05 cells showed the most robust response to *KRAS* suppression. These four responsive cell lines showed at least 5-fold more DM CpGs compared to the seven other pancreatic cell lines tested (Fig. 2C and Table 1). Although Pa16C cells are derived from Panc 10.05 cells^33^, Pa16C cells had more than 4-fold the number of DM CpGs (Fig. 2C **and** Table 1). The four responsive cell lines showed a significant number of DM CpGs located in the promoter region (200–1500 nt upstream of the transcription start site) of dozens of functionally important genes (Table 2). The methylation changes associated with *KRAS* suppression appeared to be cell line specific and were not generalizable within pancreatic cell lines. Although the methylation patterns in the NS shRNA-treated control cells were similar (Fig. 1), each cell line responded differently to the depletion of *KRAS*. Furthermore, two distinct groups emerged from the pancreatic cell lines, with seven lines displaying significantly less differential methylation compared to the four responsive cell lines (Pa16C, Pa01C, PANC-1, and Panc 10.05 cells). Taken together these results suggest that depleting oncogenic KRAS expression is cell line specific but also stochastic in nature. It is possible that whether a cell’s CpG methylation profile is responsive or refractory to *KRAS* suppression likely depends on its genetic background and other factors.

**Table 1:** Mutation status of crucial genes and the total number of differentially methylated (DM) CpGs with Δβ value ≥0.2 or ≤−0.2 in KRAS-depleted pancreatic cancer cell lines.

Cell Line	KRAS	CDKN2A	TP53	SMAD4	Hypermethylated Promoter CpGs/total CpGs	Hypomethylated Promoter CpGs/total CpGs	All CpGs with Δβ ≥0.2 or ≤−0.2
Pa16C	G12D/WT		I255N*		434/3275	764/5613	8888
Pa01C	G12D/WT		T155P*	Del*	175/1248	393/2998	4246
PANC-1	G12D/WT	Del*	R273H*		128/717	556/3136	3853
Panc 10.05	G12D/WT		I255N/WT		59/452	275/1508	1960
Pa04C	G12V*	Del*	Del*		13/172	25/200	372
Pa02C	Q61H*	Del*	L247P*	Del*	28/185	28/184	369
CFPAC-1	G12V/WT		C242R*	Del*	7/89	12/115	204
HPAC	G12D*	Stop/Stop			16/104	15/92	196
HPAF-II	G12D/WT	Del-FS*	P151S*		14/96	5/68	164
SW-1990	G12D*	Del*			11/78	15/79	157
Pa18C	G12D/WT	Del*		Del*	6/72	6/72	144

The CpG methylation in Pa16C, Pa01C, PANC-1 and Panc 10.05 cells appears to be the most responsive to KRAS depletion. Homozygous mutations are represented with an asterisk.

**Table 2:** Categorization of differentially methylated (DM) Promoter CpGs in KRAS-inhibited most responsive cell lines (Pa16C, Pa01C, PANC-1 and Panc 10.05 cells).

Pa16C cells					Pa01C cells			PANC-1 cells			Panc 10.05 cells		
Hyper- methylated		Hypo- methylated			Hyper- methylated	Hypo- methylated		Hyper- methylated	Hypo- methylated		Hyper- methylated	Hypo- methylated	
**Transcription Factors**													
ASCL2	PFDN5	ARNT	HOXD8	SMARCA5	ALX1	ALX4	PAX7	ID3	AIRE	LSR	ASCL1	BNC2	LHX4
CBX4	RAX	ATOH7	INSM2	TAF3	ALX3	BARHL2	PDX1	KLF14	CUX2	MAF	BRF1	C13orf15	LIN28A
CRIP1	RING1	BACH2	IRF7	THRB	EZH1	BHLHE22	PHOX2A	MLLT6	EBF4	NEUROG1	MKX	CASZ1	LYL1
E2F2	RREB1	BAZ2B	IRX1	TMF1	HMGB2	BNC1	PHOX2B	MSX2	EGR3	NFATC4	NEUROG1	CBFA2T3	MSC
EBF4	SALL4	BRF1	IRX2	TRIM13	HMX2	BRF1	PITX2	NKX2-5	EMX1	NKX2-6	ZFP30	ESR2	MSX1
ELK3	SIX3	BTF3	LIN28A	TRIM27	HOXB1	DBX1	PLAGL1	NKX3-1	ETV7	NKX6-2		EVX2	PAX7
EOMES	TCF7	CECR6	LMX1B	TSC22D2	IRX1	DBX2	PRDM13	PAX1	FOXA2	NRIP1		FEZF2	PCGF3
EYA2	TLX2	CNPY3	MED24	TSHZ3	KDM3B	DLX1	RNF2	PRDM8	FOXB1	PBX4		FOXE3	RARG
FOXC2	TUB	CSRP1	MIXL1	TWIST1	LHX8	DMRT1	RORB	TBX2	FOXD3	PER1		FOXI1	RUNX3
FOXE1	TULP1	CSRP2	MKX	ULK2	NEUROG1	DMRTA2	RUNX3	TCF7L2	FOXF1	PHF11		GBX2	SALL1
GBX1	UNCX	CUX2	MSX2P1	UTF1	NKX2-2	ESR1	SALL3	YAF2	GATA5	PITX3		GSC	SALL3
GSX1	VENTX	ELK4	NCALD	VAX1	PAX3	EYA4	TBX3	ZNF213	GFI1	POU3F1		HKR1	T
HAND1	ZAR1	EN1	NEUROG1	VSX1	SOX8	FEZF2	TCF4	ZNF222	GSC2	RARA		HOXA9	TLX3
HAND2	ZBTB16	ERMP1	NEUROG3	YBX2	TLX2	FOXB1	ULK2		GSX1	RAX		HOXB13	TUB
HES2	ZFP28	ESR1	NFYC	ZBTB22	TWIST1	GBX2	ZIC1		HAND1	RORB		HOXB2	VEZF1
HNF1A	ZSCAN12	ESRRG	OLIG1	ZFP30	ZNF213	GCM2	ZNF16		HAND2	SIM2		HOXB4	ZBTB16
HOXB1		EYA4	PDLIM5	ZIC1	ZNF593	GFI1	ZNF18		HES4	SIX2		HOXB8	ZBTB17
HOXC10		FOXE3	PHOX2A	ZMYND11		HIC1	ZNF256		HEYL	TBX5		IRX2	ZFP28
HOXC4		FOXG1	PLAGL1	ZNF124		HOXA6	ZNF331		HLX	THRB		IRX3	ZIC1
HOXD12		GBX2	POU3F2	ZNF135		HOXA9			HMX3	TOX		LEF1	ZNF236
HOXD3		GLI3	POU4F1	ZNF18		HOXB13			HNF1A	VENTX			
HOXD4		GRHL1	PPARG	ZNF207		HOXB2			HNF1B	WT1			
HOXD9		HIF3A	PRDM13	ZNF211		HOXC9			HOXC13	YBX2			
IRF4		HMX3	PRDM14	ZNF219		HOXD3			HOXD1	ZAR1			
KCNIP3		HOXA5	PRDM6	ZNF232		ID4			HR	ZFP37			
MYCNOS		HOXA9	RARG	ZNF268		IRF7			IRF6	ZNF229			
NEUROG2		HOXB13	RBBP9	ZNF295		MKX			IRF8	ZNF334			
NFIC		HOXB3	RUNX3	ZNF318		MSC			ISL1	ZNF701			
NKX6-1		HOXB4	SALL1	ZNF532		MSX1			LEF1	ZSCAN12			
NKX6-3		HOXB8	SALL3	ZNF682		NKX2-5							
OSR1		HOXC8	SAMD4B			NKX6-2							
OTP		HOXD1	SIM2			PAX1							
**Cytokines & Growth Factors**													
BDNF	LEFTY1	BMP3	FGF2	NRG3	FGF20	CALCA	GRP	CXCL5	BMP2	LTBP2	CALCA	CMTM2	SCT
CALCA	LTBP3	CCK	FGF20	PTHLH	GDF6	CMTM2	HAMP	KL	BMP7	MDK	CCK	FGF2	SEMA5A
CSF1	PDGFRA	CMTM1	FGF5	SEMA6D	IL28B	EPO	NGF		BMP8A	NGF		GRP	SLIT1
CXCL12	PENK	CMTM3	FGF9	SLIT1	NRG3	FGF11	PSPN		CXCL16	NRG1		NPY	
FGF19	PTH2	DKK1	GDNF	TNFSF13	PDGFA	FGF12	SEMA5A		EDN3	NRG3			
GDF7	SCGB3A1	EPO	GREM1		SEMA6B	FGF2	SLIT1		FAM3B	RLN3			
KL	SECTM1	FGF11	KITLG			GREM1	SLIT2		FGF22	STC2			
									FGF6	TNFSF12			
									GDF10	TYMP			
									GDF7	VEGFC			
									GRP				
**Homeodomain Proteins**													
GBX1	NKX6-1	CUX2	HOXC8	POU3F2	ALX1	ALX4	HOXD3	MSX2	CUX2	ISL1	MKX	EVX2	HOXB8
GSX1	NKX6-3	EN1	HOXD1	POU4F1	ALX3	BARHL2	MKX	NKX2-5	EMX1	NKX2-6		GBX2	IRX2
HNF1A	OTP	GBX2	HOXD8	TSHZ3	HMX2	DBX1	MSX1	NKX3-1	GSC2	NKX6-2		GSC	IRX3
HOXB1	RAX	HMX3	IRX1	VAX1	HOXB1	DBX2	NKX2-5		GSX1	PBX4		HOXA9	LHX4
HOXC10	SIX3	HOXA5	IRX2	VSX1	IRX1	DLX1	NKX6-2		HLX	PITX3		HOXB13	MSX1
HOXC4	TLX2	HOXA9	LMX1B		LHX8	GBX2	PAX7		HMX3	POU3F1		HOXB2	PAX7
HOXD12	UNCX	HOXB13	MIXL1		NKX2-2	HOXA6	PDX1		HNF1A	RAX		HOXB4	TLX3
HOXD3	VENTX	HOXB3	MKX		PAX3	HOXA9	PHOX2A		HNF1B	SIX2			
HOXD4		HOXB4	MSX2P1		TLX2	HOXB13	PHOX2B		HOXC13	VENTX			
HOXD9		HOXB8	PHOX2A			HOXB2	PITX2		HOXD1				
						HOXC9							
**Protein Kinases**													
CAMK2B	PDGFRA	AATK	MAPK7	PINK1	CDK6	CDKL3		BRAF	ACVR1C	KDR	PRKAA2	CDC42BPB	NEK3
FASTK	STK19	BRAF	MYO3A	SGK1	DDR1	DCLK2		CSNK1A1	BCR	KSR2	RIPK3	FGFR1	NTRK3
HUNK	TNK2	CDC42BPB	NEK10	SNRK	EIF2AK2	NEK9			CDKL2	MAST4			
KDR		CDKL3	NEK3	STYK1	HIPK3	PDK2			CSNK1G2	MST1R			
LCK		FGFR1	NRBP1	ULK2	MAP2K1	PINK1			DAPK1	PBK			
MAP3K6		FGR	NTRK3		MAPK4	RIOK3			DMPK	STK32A			
MAPK12		FYN	PBK		MATK	ULK2			EPHA6	STK32B			
					PRKD1				FGFR1	STK33			
					RYK				FLT3	SYK			
									HCK	TNK1			
									HUNK	WNK2			
									INSR				
**Oncogenes**													
CCND2	ZBTB16	ARNT	GAS7	PPARG	CCND2	HOXA9		BRAF	BCR	KDR		CBFA2T3	NTRK3
IRF4		BRAF	GNAS	TOP1	CDK6	PAX7		CCND1	CDH11	MAF		FGFR1	PAX7
KDR		ELK4	HOXA9	TRIM27	JAK2	ZNF331		MLLT6	DDX6	PER1		HOXA9	TLX3
LCK		FGFR1	HSP90AB1		JAK3				FGFR1	RARA		LYL1	ZBTB16
PDGFRA		FIP1L1	NTRK3		PAX3				FLT3	SEPT9			
					TCL1A				HOXC13	SYK			
**Cell Differentiation Markers**													
CD40	PROM1	ADAM17	IL17RA	TNFRSF10B	DDR1	CD248		PVRL2	CDH1	INSR		FGFR1	TNFRSF1B
CD81		FGFR1	ITGB3	TNFRSF8	NCAM1	CDH2			CDH2	KDR		IFITM1	
IL10RA		FZD10	MME	TNFSF13	TNFRSF8				FGFR1	LAMP3			
KDR		IFITM1	NGFR						FLT3	MME			
PDGFRA		IGF2R							FZD10	MST1R			
									GP1BB	THBD			
									ICOSLG				
**Tumor Suppressors**													
EXT2		BRCA1				PHOX2B			BRCA1	WT1		FANCA	
HNF1A									CDH1	XPA			
									HNF1A				

### Inhibitor treatment shows limited role for ERK in differential CpG methylation of Pa16C pancreatic cancer cells

Next, we investigated whether the methylation changes associated with *KRAS* suppression are dependent on ERK signaling, a major downstream effector of KRAS. We used Pa16C cells, the cell line with the greatest number of DM CpGs associated upon *KRAS* suppression (Fig. 2C), to test the effects of ERK inhibition on DNA methylation. Pa16C cells were treated with the ERK1/2-selective inhibitor, (ERKi, SCH772984)^34^ (Supplementary Fig. S5) and the cells were harvested for protein and genomic DNA 3 and 7 days after treatment. The dose response of SCH772984 on Pa16C cell growth was determined (Supplementary Fig. S5A). Based on this, Pa16C cells were treated with 0.25 μM (3.6 on log scale), which resulted in the highest inhibition of cell growth. ERKi treatment led to growth arrest as evidenced by the lower cell confluency compared to DMSO control (Supplementary Fig. S5B**, Right**) and also reduced total ERK protein and phosphorylated ERK protein as measured by western blot (Supplementary Fig. S5B**, Left**). Three and 7 days of ERKi treatment resulted in 29 and 37 DM probes, respectively. Only 1 CpG probe cg18988094 was hypomethylated in both 3- and 7-day ERKi-treated samples. This DM CpG is found near the gene STIP1, which has been reported to activate ERK signaling (Supplementary Fig. S5C). We compared DM CpG profiles of the ERKi-treated Pa16C cells to the Pa16C cells transduced with shKRAS. However, there were no overlapping DNA methylation changes between the ERKi-treated and the KRAS shRNA-transduced Pa16C cells despite the similar effects on cell growth observed in both conditions (Supplementary Fig. S5A**, Right**)^15^. Furthermore, *KRAS* shRNA induced >8000 DNA methylation changes compared to <40 DM CpGs after pharmacological ERK inhibition. These observations suggest that targeted ERK inhibition leads to Pa16C cell growth arrest similar to the growth arrest observed in KRAS shRNA transduced Pa16C cells. However, ERK does not appear to be consequential to the thousands of KRAS-associated DM CpGs present in the *KRAS* shRNA transduced Pa16C cells, at least after 7 days and suggests that KRAS suppression leads to sustained DM changes not affected by inhibition of downstream targets like ERK. However, it remains possible that ERK could still be responsible for KRAS-associated methylation changes that occur over a longer time frame.

### Gene ontology analysis of differentially methylated promoters in KRAS-depleted pancreatic cancer cell lines

**Figure 3:**
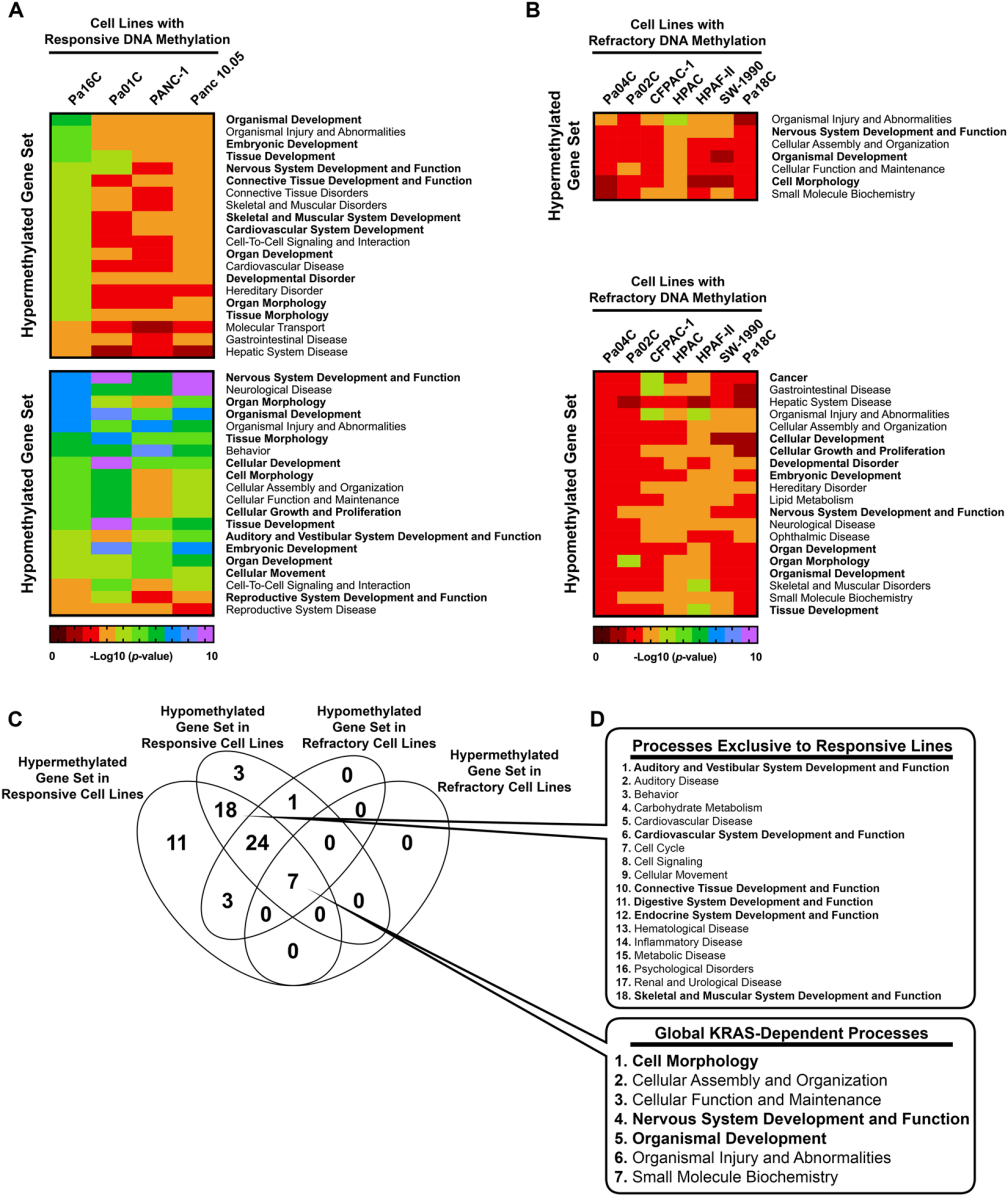
Gene ontology analysis of differentially methylated (DM) promoters in KRAS- inhibited pancreatic cancer cells. (**A**,**B**) Gene Ontology analysis of DM genes in cells with **(A)** responsive or **(B)** refractory DNA methylation. Processes related to development and differentitation are in bold. **(C)** Venn diagram showing the number of biological processes associated with responsive or refractory promoter CpG methylation in KRAS-depleted cell lines. **(D)** (Top) List of affected biological processes exclusive to cell lines responsive to KRAS-depletion, or (Bottom) common among all of the KRAS-depleted cell lines.

Due to the limited number of overlapping DM CpGs (Supplementary Fig. S4), we attempted to isolate biological processes associated with *KRAS* knockdown that are common between KRAS-depleted cell lines. First, we grouped the KRAS-depleted cell lines into “responsive” cells (Pa16C, Pa01C, PANC-1, and Panc 10.05 cells) and “refractory” cells referring to the other seven pancreatic cell lines in our panel, based on the number of DM CpGs identified (Fig. 2C). To focus our analysis on genes with DM CpGs most likely to produce transcriptional effects, we isolated DM CpGs found within promoter regions, 200–1500 bases upstream of the transcription start site of a gene, and within 4 kb of a CpG island, including shores and shelves. We then kept only the gene promoters that had consistent CpG differential methylation, where all of the CpGs were either hypermethylated or hypomethylated. Genes encoding transcription factors, oncogenes, kinases, and growth factors showed differential DNA methylation at their promoters in KRAS-depleted cells (Table 2). Gene ontology analysis was performed using lists of promoters from each KRAS-depleted cell line that were hypermethylated or hypomethylated. The top ≤ 20 overlapping biological processes were compiled in a heat map (Fig. 3). Hypermethylated promoters in the responsive cells were enriched for genes involved in development and differentiation (Fig. 3A**, bold**); however, the number of hypermethylated promoters was significantly reduced in the refractory cells (Table 1), which limited the number of associated biological processes (Fig. 3B). The hypomethylated promoters in both the responsive cells and the refractory cells were enriched for genes involved in development and differentiation (Fig. 3A,B). Gene ontology analysis produced a significantly lower number of biological processes for the refractory cell lines compared to responsive cells due to the paucity of DM CpGs in the these cell lines (Fig. 3C). A total of 18 biological processes were found exclusively in the responsive lines with 6 of these related to development (Fig. 3D**, Top, bold**). In addition, our analysis showed 7 processes that were potentially affected by *KRAS* suppression in both responsive and refractory cell lines (Fig. 3D**, Bottom**). Together these results suggest that *KRAS* suppression leads to differential DNA methylation affecting genes involved in development and differentiation, especially in responsive cell lines, and corroborates previous gene ontology analyses of DM genes in HRAS-transformed fibroblasts, which also showed an enrichment for genes involved in development and differentiation^28^.

### DNA methylation changes associated with mutant KRAS overexpression in lung cells

**Figure 4:**
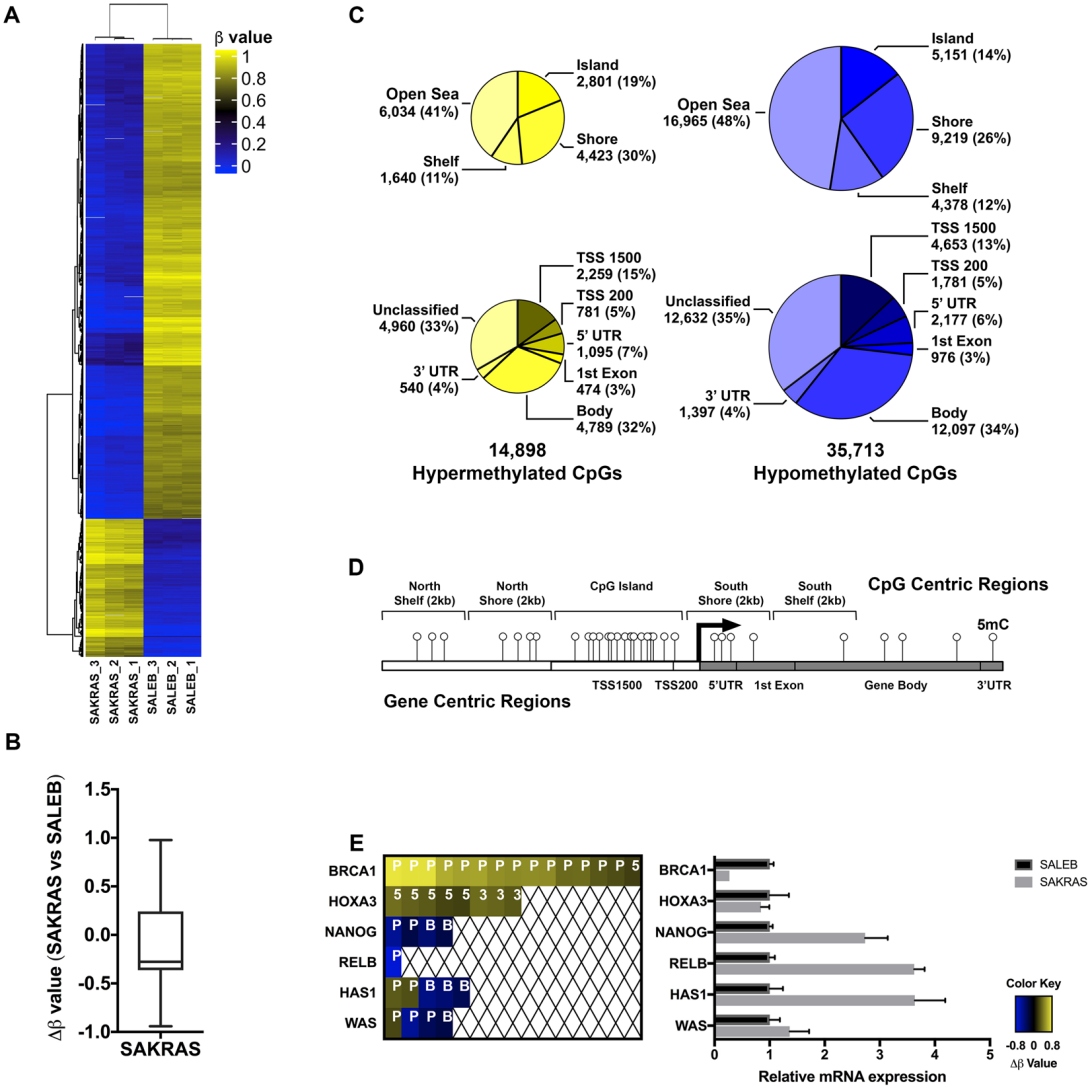
DNA methylation changes associated with mutant KRAS overexpression in SALEB lung cells. (**A**) Hierarchical clustering of the top 1000 differentially methylated probes for SALEB and SAKRAS cell lines. **(B)** Box plot showing overall delta β vales (median of −0.27664) in the SAKRAS cells compared to SALEB cells. **(C)** Annotation of hypermethylated (left; yellow) and hypomethylated (right; blue) CpGs to CpG islands (top) and gene functional regions (bottom). **(D)** Diagram showing examples of CpG centric and gene functional centric regions analyzed by the Infinium DNA methylation array. **(E)** Genes of interest with DM CpGs in SAKRAS cells. Each colored block represents one DM CpG at the respective region of the stated gene. P, promoter region, 5, 5’UTR; B, Body, gene body; 3, 3′UTR (left panel); The mRNA expression of these genes was measured using qRT-PCR (right panel).

Since our results indicate that *KRAS* suppression is associated with CpG methylation changes in pancreatic cancer cell lines, we hypothesized that the overexpression of oncogenic KRAS would also lead to DNA methylation changes. To isolate the effects of oncogenic KRAS overexpression, we used an isogenic lung model for this experiment and performed the experiment in triplicate. KRAS is mutated in approximately 30% of all lung cancers^35^, making lung cells a relevant model to study the effects of activating KRAS mutations. We surveyed the CpG methylation patterns in low passage, immortalized lung epithelial cells stably expressing exogenous KRAS G12V (SAKRAS cells) and compared these cells to non-transformed empty vector controls (SALEB cells). Our analysis showed significantly greater DM CpGs in SAKRAS lung cells overexpressing KRAS G12V (50,611 DM CpGs) compared to Pa16C pancreatic cells with KRAS knockdown (8,888 DM CpGs). Compared to non-transformed SALEB cells, SAKRAS lung cells overexpressing KRAS G12V displayed significantly greater hypomethylated CpGs (Fig. 4A,B). Further categorization of the DM CpGs into “CpG centric” (Fig. 4C**, top**) and “gene centric” (Fig. 4C**, bottom**) regions reveal the postional and functional distribution of the methylation changes associated with KRAS G12V overexpression (Fig. 4C,D). The effects on mRNA expression corresponding to six genes of interest haboring DM CpGs was measured using qRT-PCR (Fig. 4E). Promoter hypermethylation correlated with reduced mRNA expression of *BRCA1*, and hypomethylation correlated with increased expression of *NANOG* and *RELB*. However, the relationship between promoter methylation and transcription was not directly correlated in other genes (Fig. 4E). Although changes at individually important CpGs may alter gene expression, alterations to an entire CpG region may be better correlated with changes in gene expression (Fig. 4E**, BRCA1**). Taken together, these data indicate that overexpression of oncogenic KRAS G12V is associated with significant CpG methylation changes in SALEB cells.

### Gene ontology analysis of DM CpGs reveals enrichment of genes involved in development and differentiation associated with changes in KRAS expression

**Figure 5:**
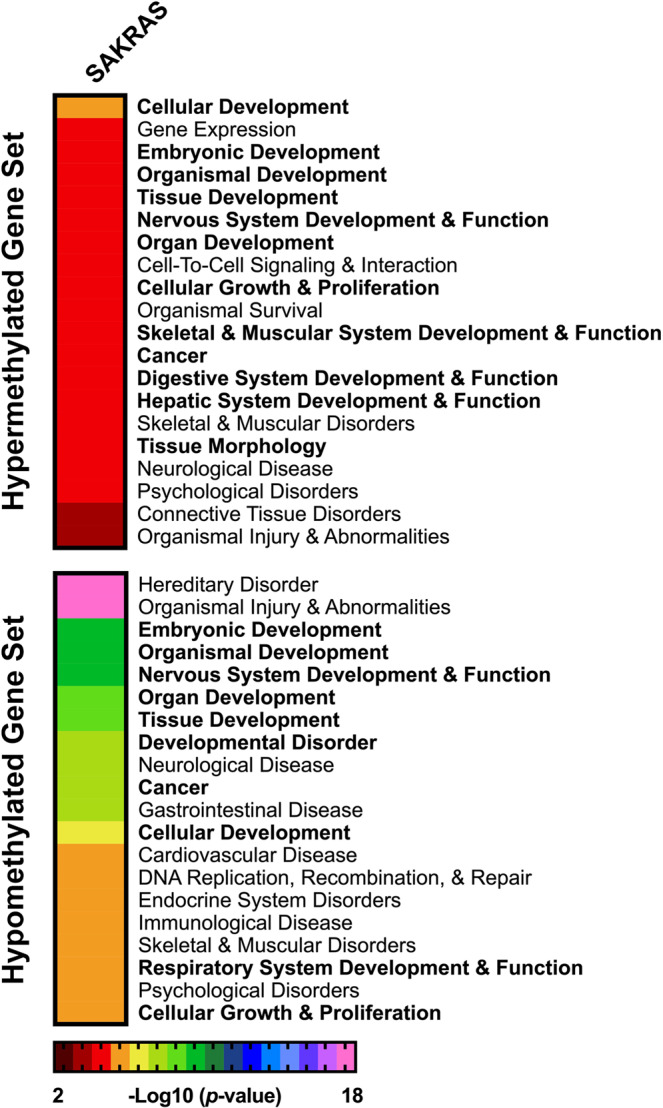
Gene ontology analysis of differentially methylated promoters associated with KRAS G12V overexpression in lung cancer cells. Gene ontology analysis of the hypermethylated (Top) and hypomethylated (Bottom) gene sets from the SAKRAS lung cell line are ranked using a negative log10 scale of the p-values. The top 20 biological processes are shown. Biological processes involved in cell development and differentiation shown in bold.

To focus our analysis on genes with DM CpGs most likely to produce transcriptional effects in SAKRAS cells overexpressing KRAS G12V, we isolated promoter regions with consistently DM CpGs as previously described for the pancreatic cell lines (Fig. 3). Five hundred and forty-seven genes met these conditions, including 196 genes with hypermethylated promoters. Genes encoding transcription factors, oncogenes, kinases, and growth factors showed differential DNA methylation at their promoters in SAKRAS cells overexpressing KRAS G12V (Table 3). Gene ontology analysis using the list of 196 hypermethylated gene promoters, and 351 hypomethylated gene promoters in SAKRAS cells overexpressing KRAS G12V, showed an enrichment for genes involved in development and differentiation (Fig. 5), consistent with our previous mutant KRAS loss-of-function studies performed in pancreatic cancer cells (Fig. 3 **and** Supplementary Fig. S6D,E). Gene ontology analysis using the list of hypermethylated and hypomethylated gene promoters from both SAKRAS KRAS G12V expressing cells and Pa16C KRAS knockdown cells, showed the common enrichment for genes involved in differentiation and development (Supplementary Fig. S6D,E). It is noteworthy that while mutant KRAS knockdown and overexpression ultimately results in DM CpGs of genes involved in similar biological processes, the specific number of genes and location of DM affected are distinct and unique to each cell line.

**Table 3:** Categorization of gene promoters with differentially methylated (DM) CpGs associated with KRAS overexpression in SAKRAS lung cell line.

SAKRAS cells			
Hyper- methylated		Hypo- methylated	
**Transcription Factors**			
BARHL2	HOXA5	AFF2	OTX1
BARX2	IRX1	BRDT	PAX2
C11orf9	IRX3	CITED1	PAX9
CDX1	KEAP1	CRIP1	SALL1
CDX2	KLF11	ELF4	SIM2
CTNNB1	MYBL2	EMX1	SNAPC2
ETV7	NKX2-3	FOXC2	SOX1
FEZF2	NKX6-2	FOXG1	SOX11
FHL2	PAX7	FOXO4	SOX3
FOXA2	POU3F2	GSC	TAF1
GATA5	PRDM2	HEYL	TBX1
GBX2	SOX21	HIC1	TBX2
HAND1	TBX3	HOXA9	TBX4
HES5	ULK2	HSF4	TLX2
HES6	UNCX	ISL2	TSC22D3
HHEX	UTF1	LHX2	ZFP161
HNF1B	VAX1	LMX1A	ZIC3
HOXA2	ZIM2	NFYB	ZNF132
		NKRF	ZNF318
		OLIG2	ZNF630
**Cytokines & Growth Factors**			
APLN		ADM2	NGF
CMTM2		EDN3	NPY
FGF22		FGF13	OXT
NPPC		GAL	STC2
PYY		GDF7	
**Homeodomain Proteins**			
BARHL2	IRX1	EMX1	
BARX2	IRX3	GSC	
CDX1	NKX2-3	HOXA9	
CDX2	NKX6-2	ISL2	
GBX2	PAX7	LHX2	
HHEX	POU3F2	LMX1A	
HNF1B	UNCX	OTX1	
HOXA2	VAX1	PAX2	
HOXA5		TLX2	
**Protein Kinases**			
CSNK1D		BRDT	MST4
EPHA8		CDKL5	PDK3
FLT1		FASTK	RPS6KA3
GUCY2D		IRAK3	TAF1
STK32C		MAPK4	
ULK2			
**Oncogenes**			
CDX2		ELF4	MSI2
CTNNB1		FOXO4	OLIG2
FEV		GNAS	SEPT9
PAX7		HOXA9	TCL1A
**Cell Differentiation Markers**			
FUT4		CD151	
GP1BB		CD8A	
		IL13RA1	
		PTPRJ	
**Tumor Suppressors**			
BRCA1		FAM123B

To directly assess the role of mutant KRAS in maintaining DNA methylation patterns in the isogenic lung cells, we identified differentially methylated (DM) CpGs from SAKRAS cells in which KRAS expression had been suppressed transiently with KRAS siRNA and compared these to cells transfected with control siRNA **(**Supplementary Fig. S6A,B). We observed 86 DM CpGs in SAKRAS cells following siRNA-mediated KRAS knockdown (Supplementary Fig. S6B). Interestingly, only two of these CpGs were also DM in the SAKRAS vs SALEB cell comparison. This included LRRC7 and the pluripotency transcription factor, NANOG, which were both hypomethylated in SAKRAS cells compared to SALEB cells, and then hypermethylated following KRAS depletion via siRNA (Supplementary Fig. S6B**, Left**). We identified 10 probes that were differentially methylated in opposite directions when comparing the Pa16C cells in which KRAS had been depleted with shRNA to the SAKRAS cells (Supplementary Fig. S6C). Taken together, the lists of DM genes affected by changes in KRAS expression while distinct between cell lines, showed an enrichment for genes involved in development and differentiaion.

## Discussion

The RAS small GTPase is the most commonly mutated oncoprotein in cancer^1^. RAS and its downstream effectors control key aspects of cancer development. However, until recently, attempts to directly target oncogenic KRAS have been unsuccessful. In addition to aberrant signaling, the expression of mutant KRAS is correlated with global differential DNA methylation^24,25^. Therefore, epigenetic changes associated with oncogenic KRAS expression could be an avenue where the survival of KRAS-dependent cancer cells may be vulnerable. Here we demonstrated that the that cell type was more impactful than mutant KRAS on DNA methylation. KRAS-mutant PDAC cell lines were also classified based on the responsiveness of their methylome to KRAS depletion. Furthermore, a number of studies suggest that the majority of differential DNA methylation associated with cancer may be stochastic in nature - contributing to low levels of overlap and high heterogeneity between cell lines, even when they share the same genetic background and/or origin^28,36–40^. It is most likely due to this stochastic nature that we did not observe previously described methylation events driven by RAS, such as the silencing of proapoptotic FAS by HRAS in fibroblasts^24^, and the silencing of IRAK3 by mutant KRAS^21^. However, we did identify novel changes in genes related to development and differentiation after KRAS silencing, which was common to all our pancreatic cancer cell lines but was more pronounced in the KRAS-responsive lines. This suggests that while many DNA methylation changes could be stochastic in nature and simply “passenger” events, or a consequence of their cell state and cell lineage, KRAS is likely still able to influence key changes to the epigenome that are ultimately crucial for the cancer phenotype. More studies are needed to determine whether stratification, such as by cancer subtype, will reveal more consistent changes in methylation patterns.

**Figure 6:**
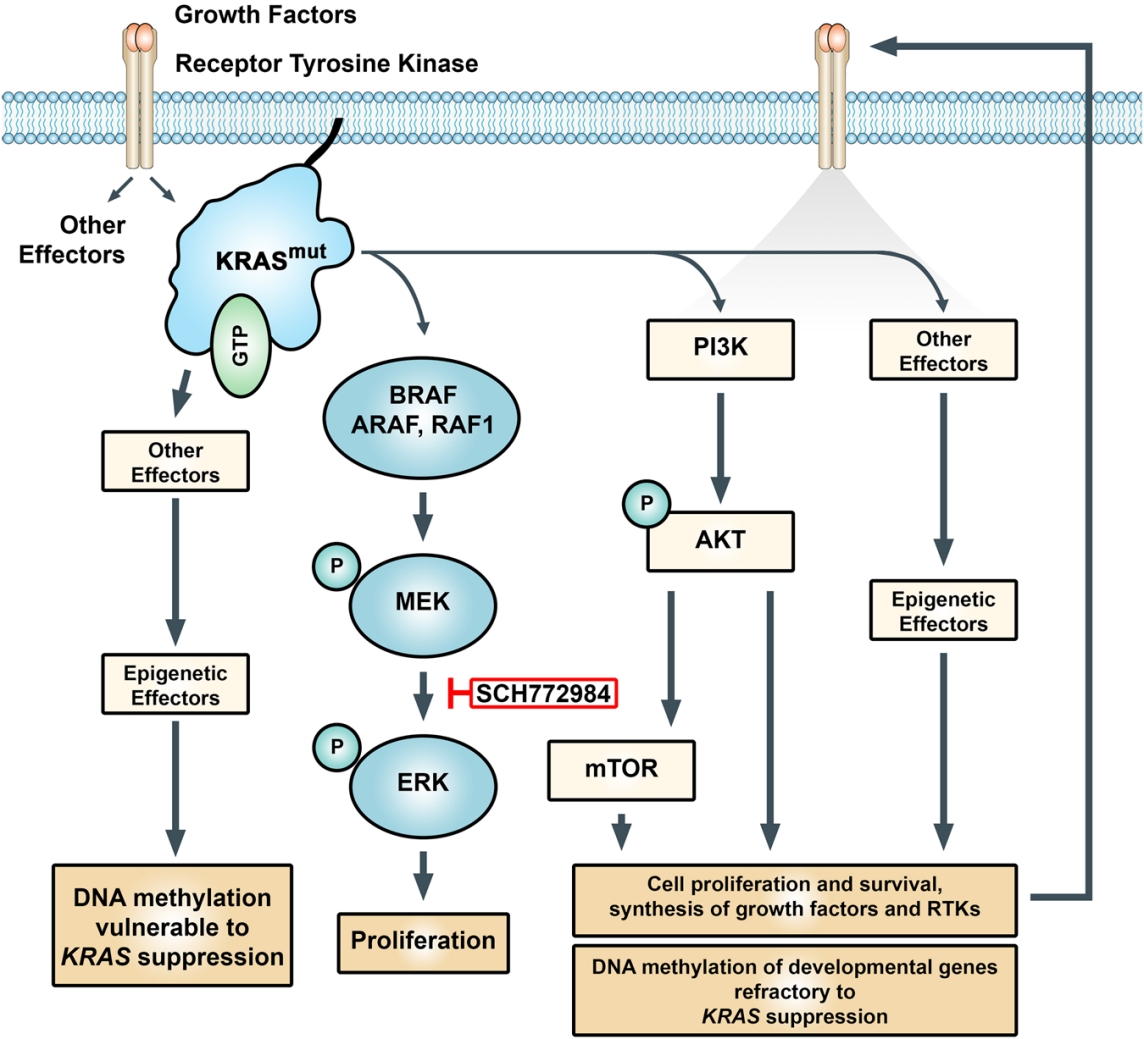
Model showing epigenetic regulation of developmental genes by mutant KRAS. Activating KRAS mutations lead to persistant induction of effector pathways that drive the cancer phenotype including the differential DNA methylation of genes involved in development and differentiation. In some cell lines, effector pathways such as PI3K and others, are able to maintain their abberant activity independent of KRAS signaling. As a consequence of feed forward loops initiated by mutant KRAS, kinome reprogramming, or the establishment of stable epigenetic patterns, the majority of DNA methylation changes associated with mutant KRAS activity remains refractory to KRAS suppression. However, independent of the changes in DNA methylation, KRAS knockdown and ERK inhibition still both lead to growth arrest in KRAS driven cell lines. SCH772984, type I and type II ERK inhibitor.

Another interesting observation is the variable number of DM CpGs associated with *KRAS* knockdown and/or KRAS overexpression. KRAS remains crucially linked to cell proliferation through RAF-MEK-ERK mitogen activated protein kinase (MAPK) cascade, its main effector pathway, and inhibition of this pathway reliably leads to growth arrest^15^. However, we showed that ERK was not responsible for changes in the methylome, at least over the time frame observed. While it is possible that ERK could play a role in methylation changes over a longer period of time, the question remains, if not ERK, which KRAS effectors are leading to short term DNA methylation changes. In mouse lung adenocarcinoma cells, YAP1 was able to rescue KRAS depleted cells, suggesting a relevant mechanism to bypass loss of KRAS signaling^41^. In the same study, KRAS also induced PI3K expression, and yet, the subsequent suppression of KRAS has no effect on the upregulated AKT activation. PI3K has been shown to compensate for *KRAS* suppression in pancreatic cancer cells and regulate epigenetic modifiers including DNMTs^42^. Cells in which *KRAS* levels have been genetically reduced display sensitivity to PI3K inhibitors and dual PI3K and MEK inhibitors have been found to be more effective than blocking the individual pathways alone^43^. PI3K/AKT signaling has been shown to be an epigenetic regulator in multiple cancers by modulating the activity of DNA methyltransferase I (DNMT1)^44^. It is possible that persistent PI3K-AKT activation, even after *KRAS* suppression, may be able maintain the majority of methylation changes induced by mutant KRAS. This kind of sustained activity by effector pathways could maintain the methylation status of the majority of the changes initially induced by mutant KRAS expression, but were not reversed upon KRAS knockdown (Fig. 6).

DM CpGs associated with KRAS overexpression in our study have been localized to the promoters of important tumor suppressors, oncogenes, transcription factors, and regulators of differentiation, with gene ontology analysis revealing an enrichment for differentially methylated genes involved in differentiation and development. The regulation of pluripotency and lineage-specific genes requires the integration of multiple signaling pathways, epigenetic modifiers, and transcription factors^45^. In response to *KRAS* suppression, KRAS-driven cells may rely on compensatory survival pathways such as the PI3K pathway. PI3K-AKT has been shown to affect the expression of differentiation and stemness genes. In our pancreatic cells, particularly KRAS-responsive cells such as Pa16C, we identified many differentially methylated genes associated with stemness following KRAS knockdown, suggesting that KRAS could be involved in inducing stemness in cancer cells through PI3K/AKT. This includes promoter hypomethylation upstream regulators of AKT signaling, such as FGF9^46,47^, and NRG3, a ligand that activates HER3, an EGFR member of receptor tyrosine kinase (RTK) signaling upstream of PI3K-AKT^48^. POU3F2 and OLIG2 were both hypomethylated - two out of the four genes, from a core set of neurodevelopmental transcription factors (POU3F2, SOX2, SALL2, and OLIG2) essential for GBM propagation. These transcription factors coordinately bind and activate regulatory elements sufficient to fully reprogram differentiated GBM cells into tumor propagating stem-like cells^49^. Another promoter hypomethylated upon *KRAS* knockdown is HOXA9, a major transcription factor that regulates stem cells during development. Aberrant expression of HOX genes occurs in various cancers, and HOXA9 transcriptomes are specifically associated with cancer stem cell features^50^. Hypomethylation was also found at the BMP3 promoter. BMPs are implicated in activation of signaling pathways that drive epithelial-mesenchymal transition (EMT), including WNT signaling, TGFB signaling and PI3K signaling, all important pathways in pancreatic cancer cells^41,51,52^. And finally, another promoter which appeared as hypomethylated was TWIST1, a canonical EMT transcription factor shown to promote cancer stem cell properties^53^. Overexpression of TWIST1 is reported to override Myc-induced apoptosis in tumor cells and along with the other changes, could be a compensatory response by the Pa16C KRAS-mutant pancreatic cells to survive KRAS suppression.

Together, our findings suggest that while oncogenic KRAS-associated DNA methylation changes may be stochastic in nature and superseded by cell type, the changes nevertheless converge on biological processes most notably involving pathways of development and differentiation. That ERK inhibition was not analogous to KRAS suppression in Pa16C cells suggests that KRAS-mediated DNA methylation are sustained independent of ERK. Taken all together, KRAS-mediated DNA methylation are stochastic and independent of canonical downstream effector signaling. This may therefore represent a non-canonical mechanism for enhancing tumorigenic potential and possibly help explain the ineffectiveness of KRAS effector inhibition in the clinic. Exploring the KRAS-mediated methylation changes in these pathways may be a deserving direction toward identifying supplementary strategies to target KRAS-driven cancers.

## Methods

### Cell culture

PDAC cell lines were obtained from ATCC and were maintained in Dulbecco’s Modified Eagle Medium supplemented with 10% fetal calf serum (FCS) (HPAC and PANC-1), in RPMI 1640 supplemented with 10% FCS (CFPAC-1, HPAF-II, Panc 10.05, and SW-1990). Low passage SALEB and SAKRAS cells were generous gifts from Dr. Scott H. Randell (UNC-Chapel Hill) and were grown as described previously^54^. The SALEB cells were generated by infecting small airway lung epithelial cells with an amphotropic retrovirus that transduces SV40 ER, which encodes both the LT and small t antigens, and a neomycin drug resistance marker. These cells were subsequently infected with a retrovirus vector that transduces the hTERT gene together with the hygromycin resistance marker. Expression of these genetic elements was sufficient to immortalize the SALEB* cells. Finally, SALEB* cells were infected with retrovirus that transduces (i) the puromycin resistance marker (SALEB) or (ii) mutant KRAS G12V oncogene together with the puromycin resistance marker (SAKRAS). All other cells were maintained in Dulbecco’s Modified Eagle Medium (DMEM; Gibco) supplemented with 10% fetal bovine serum (FBS; EMD Millipore). Cell lines were used for no longer than six months before being replaced. Stable cell lines were generated by selection in 2 μg/ml puromycin.

### Western Blot reagents

Cells were lysed in 1% NP-40 lysis buffer (phosphatase and protease inhibitors from Sigma-Aldrich added fresh). Protein extracts were quantified by Bradford assay (Bio-Rad Laboratories) and analyzed by SDS-PAGE. Blot analyses were done with phospho-specific antibodies to ERK1/2 (T202/Y204) and antibodies recognizing total ERK1/2 to control for total protein expression. Antibody to KRAS4B was obtained from Calbiochem. Antibody for β-actin was used to verify equivalent loading of total cellular protein. Antibodies were purchased from Cell Signaling Techonology unless otherwise stated.

### Small molecule inhibitors

The ERK1/2-selective inhibitor SCH772984 was provided by A. Samatar (Merck). Inhibitors for *in vitro* studies were dissolved in dimethyl sulfoxide (DMSO) to yield a 10 mM or 20 mM stock concentration and stored at −20 or −80 °C, respectively.

### siRNA and shRNA transfection reagents

The following human siRNA (siGenome SMARTpool) was purchased from Dharmacon as a pool of four annealed dsRNA oligonucleotides: KRAS (L-005069–00) and non-targeting control #3 (D-001210-03). Dharmafect transfection reagent 1 was used to transfect 20–40 nM siRNA according to manufacturer’s instruction and cells were harvested 96 hours after transfection. The target sequence for the validated shRNA construct used to target KRAS was CAGTTGAGACCTTCTAATTGG. The lentivirus vector encoding shRNA targeting KRAS (TRCN0000010369) was provided by J. Settleman (Genentech). Target cells were transduced by combining viral particle-containing medium with complete media at a ratio of 1:4 in the presence of polybrene (8 μg/ml). Media were exchanged 8–10 h later and selection was initiated following 16 h incubation in complete media. Samples were collected 72–120 h after the initiation of selection.

### DNA methylation analysis

Global DNA methylation was evaluated using the Infinium HumanMethylation450 BeadChip Array using more than ~450,000 Infinium CpG probes (Illumina, San Diego, CA). 1 μg of each DNA sample underwent bisulfite conversion using the EZ DNA Methylation Kit (Zymo Research, Irvine, CA) according to the manufacturer’s recommendation for the Illumina Infinium Assay. Bisulfite-treated DNA was then hybridized to arrays according to the manufacturer’s protocol. We used GenomeStudio V2011.1 (Illumina) for methylation data assembly and acquisition. Methylation levels for each CpG residue are presented as β values, estimating the ratio of the methylated signal intensity over the sum of the methylated and unmethylated intensities at each locus. The average β value reports a methylation signal ranging from 0 to 1, representing completely unmethylated to completely methylated values, respectively. Methylation data was preprocessed using the DMRcate package^55^. Data preprocessing included background correction, probe scaling to balance Infinium I and II probes, quantile normalization, and logit transformation. A logit transformation converts otherwise heteroscedastic beta values (bounded by 0 and 1) to M values following a Gaussian distribution. Additionally, detection p-values>0.05 in 25% of samples, probes on X and Y chromosomes, and probes situated within 10 bp of putative SNPs were removed. Differential methylation analysis on logit-transformed values was performed to compare samples in IMA. Wilcox rank test was conducted between experimental and control samples and p-values were corrected by calculating the false discovery rate by the Benjamini-Hochberg method. Probes with adjusted p-values <0.05, and delta β values ≥0.2 or ≤ −0.2 to 4 significant figures are considered statistically significant and differentially methylated. The methylation data discussed in this publication have been deposited in NCBI’s Gene Expression Omnibus and are accessible at https://www.ncbi.nlm.nih.gov/geo/query/acc.cgi?acc=GSE119548. The ENCODE methylation data used in this publication were retrieved from the ENCODE Data Coordination Center and are accessible at https://www.encodeproject.org/ucsc-browser-composites/ENCSR037HRJ.

### RNA sequencing and analysis

RNA sequencing was performed as described in Bryant *et al*.^29^. Briefly, a panel of human PDAC cell lines was infected with lentiviral vectors encoding shRNA targeting *KRAS* or a scrambled control construct for 8–10 h, and then selected for 48–96 h (depending on cell line). Following selection, cells were washed twice in ice cold phosphate-buffered saline (PBS), scraped in ice cold PBS, collected by centrifugation, and flash frozen. Total RNA (50 ng) for the pancreatic cell lines was used to generate whole transcriptome libraries for RNA sequencing using Illumina’s TruSeq RNA Sample Prep. Poly-A mRNA selection was performed using oligo(dT) magnetic beads, and libraries were enriched using the TruSeq PCR Master Mix and primer cocktail. Amplified products were cleaned and quantified using the Agilent Bioanalyzer and Invitrogen Qubit. The clustered flowcell was sequenced on the Illumina HiSeq. 2500 for paired 100-bp reads using Illumina’s TruSeq SBS Kit V3. Lane level fastq files were appended together if they were sequenced across multiple lanes. These fastq files were then aligned with STAR 2.3.1 to GRCh37.62 using ensembl.74.genes.gtf as GTF files. Transcript abundance was quantified and normalized using Salmon in the unit of transcripts per million (TPM). Clustering was performed using R heatmap.2 package with Euclidean Distance and McQuitty clustering method. Binary sequence alignment/map (BAM) files of RNA-seq data is available from the EMBL-EBI European Nucleotide Archive (ENA) database - http://www.ebi.ac.uk/ena/ with accession number PRJEB25797. The data are accessible at http://www.ebi.ac.uk/ena/data/view/PRJEB25797. The sample accession number is ERS2363485-ERS2363504.

### Gene ontology analysis

The differentially methylated (DM) CpGs (i) in a promoter region (200–1500 bases upstream of the transcription start site of a gene) and (ii) within 4 kb of a CpG island (including CpGs at shores and shelves) are referred to as Promoter CpGs in this study. If a gene contains Promoter CpGs that did not all change in the same direction (all hypermethylated or all hypomethylated), that gene was excluded from analysis. Gene sets with hypermethylated or hypomethylated Promoter CpGs are loaded into Molecular Signature Database (MSigDB)^56^ (http://www.broad.mit.edu/gsea/) and members of each gene set are categorized by gene families. The gene ontology analyses were generated using IPA (QIAGEN Inc., https://www.qiagenbioinformatics.com/products/ingenuity-pathway-analysis)^57^. The gene set of interest was uploaded into IPA (Ingenuity Systems, Redwood City, CA) and the Core Analysis workflow was run with default parameters. The Core Analysis provides an assessment of significantly altered pathways, molecular networks, and biological processes represented in the samples’ gene list. The relative ranking order of biological processes were determined using a negative log10 scale of their p-values. The most enriched (top 20) biological processes with p-value <0.01 were picked. The gene sets used for analysis either contained hypermethylated Promoter CpGs only or hypomethylated Promoter CpGs only. Individual promoters with both hypermethylated and hypomethylated Promoter CpGs were excluded from gene set enrichment analysis.

## Supplementary information

Supplementary information.

Supplementary Figure S1

Supplementary Figure S2

Supplementary Figure S3

Supplementary Figure S4

Supplementary Figure S5

Supplementary Figure S6
