# Supplementary material for: Genome-wide DNA methylation analysis of KRAS mutant cell lines
Source: Sci Rep. 2020 Jun 23;10:10149. doi: 10.1038/s41598-020-66797-x (PMC7311523; doi:10.1038/s41598-020-66797-x)
Supplement: Supplementary file 1 — Supplementary information. [file 41598_2020_66797_MOESM1_ESM.docx]

**Genome-wide DNA methylation analysis of KRAS mutant cell lines**

Ben Yi Tew ^1†^, Joel K. Durand ^2†^, Kirsten L. Bryant^2^, Tikvah K. Hayes^2^, Sen Peng^3^, Nhan L. Tran^4^, Gerald C. Gooden^1^, David N. Buckley^1^, Channing J. Der^2^, Albert S. Baldwin^2^* and Bodour Salhia^1^*

**1** Department of Translational Genomics, University of Southern California, Los Angeles, CA 90033, USA

**2** Lineberger Comprehensive Cancer Center, University of North Carolina at Chapel Hill, Chapel Hill, NC 27599, USA

**3** Cancer and Cell Biology Division, Translational Genomics Research Institute, Phoenix, AZ 85004, USA

**4** Departments of Cancer Biology and Neurology, Mayo Clinic Arizona, Scottsdale, AZ 85259, USA

† These authors contributed equally to this work

***Corresponding Authors:**

Bodour Salhia

Department of Translational Genomics, University of Southern California, Los Angeles, CA 90033, USA

Phone: (323) 442-3099

Email: [salhia@usc.edu](mailto:salhia@usc.edu)

Albert S. Baldwin

Lineberger Comprehensive Cancer Center, University of North Carolina at Chapel Hill, Chapel Hill, NC 27599, USA

Phone: (919) 966-3652

Email: [abaldwin@med.unc.edu](mailto:abaldwin@med.unc.edu)


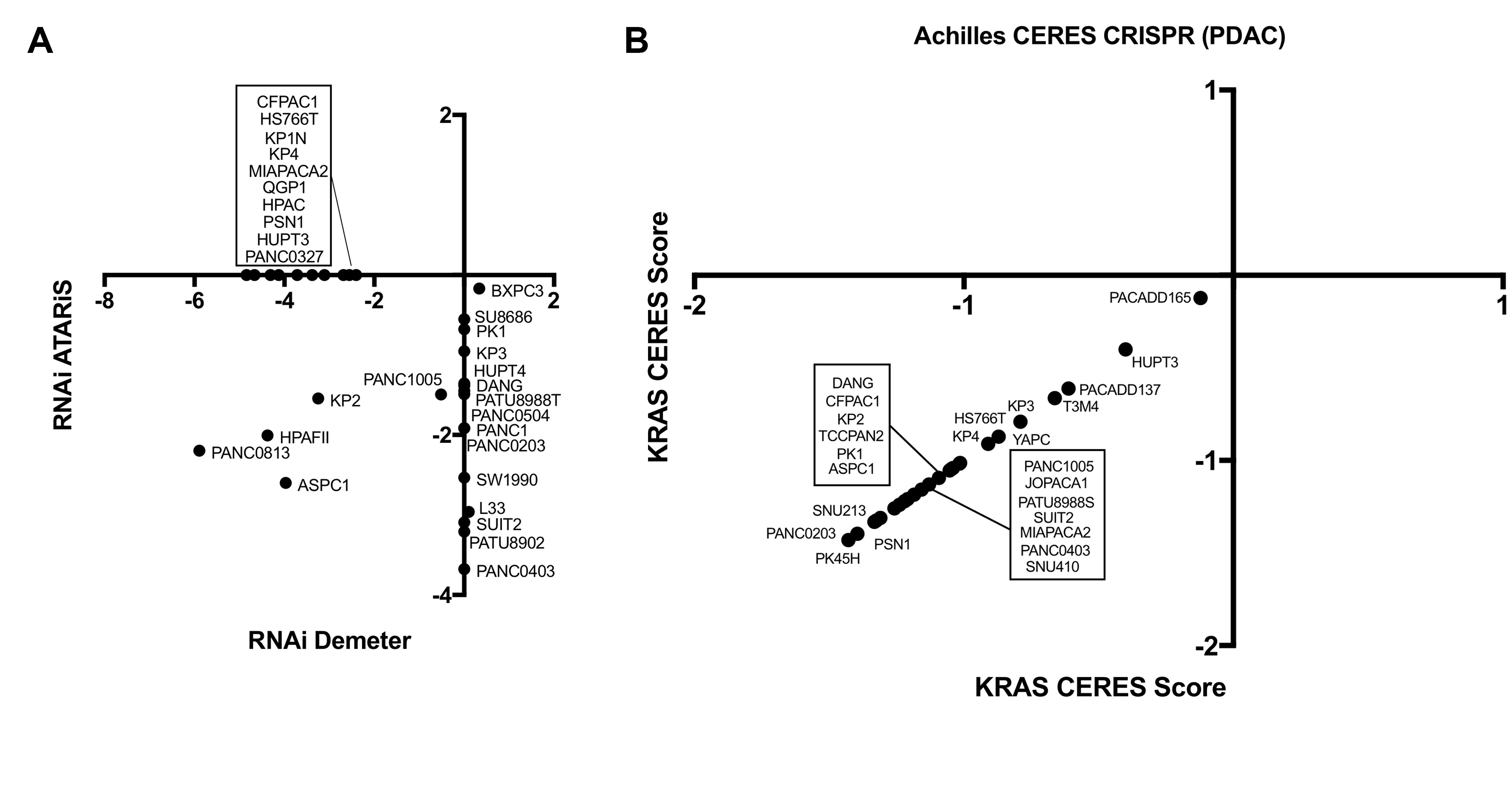


**Supplementary Figure S1. RNAi screens against KRAS showing pancreatic cell line dependence on KRAS expression.** **A.** Comparison between two RNAi datasets including 5 of the cell lines used in our methylation study; Achilles (Demeter) was performed by the Broad Institute and DRIVE (ATARiS) was performed by Novartis. The more negative, the more the cell line is dependent on KRAS expression. **B.** A CRISPR (Avana CRISPR Library) screen performed by the Broad Institute. The more negative, the more the cell line is dependent on KRAS. Plots generated using the Broad Institute Dependency Map (https://depmap.org/portal/).


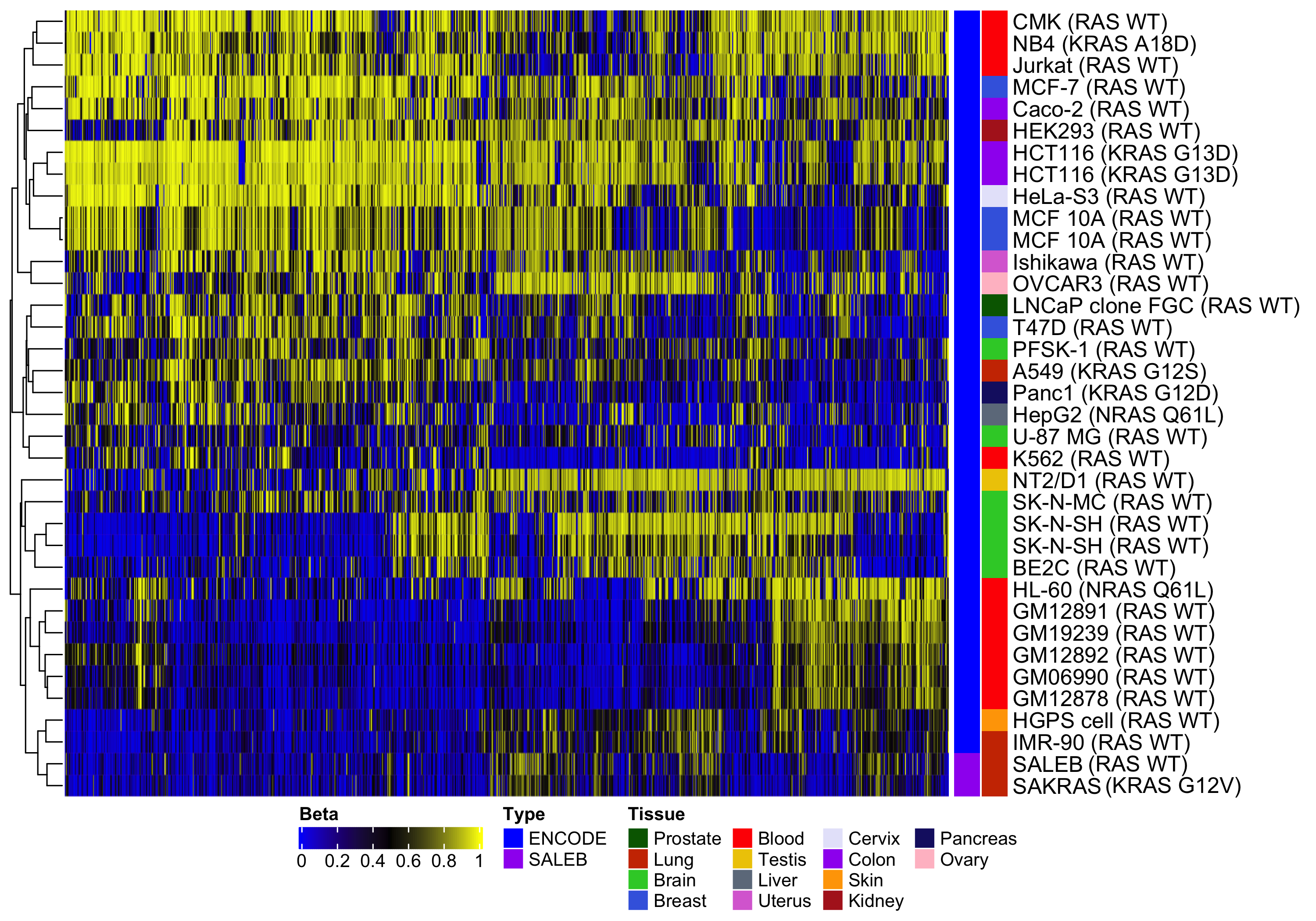


**Supplementary Figure S2. CpG methylation in a panel of cell lines with varying KRAS status, excluding transduced pancreatic cell lines.** Unsupervised hierarchical clustering analysis using the top 1000 most variable CpG probes across a panel of cell lines is displayed above. DNA methylation patterns were compared between of lung epithelial SALEB / SAKRAS cells and Infinium methylation data obtained from ENCODE (www.encodeproject.org). The β value for each probe is represented with a color scale as shown in the key. Values closer to 1 represent highly methylated CpGs, while values closer to zero represent least methylated CpGs.

**

**

**Supplementary Figure S3. GSEA analysis of genes and pathways related to KRAS-depletion in pancreatic cell lines.** **A-D.** GSEA analysis of control versus shRNA knockdown samples for each pancreatic cancer cell line. Each panel represents a different geneset associated with KRAS. The dots indicate the enrichment score for each sample, while the arrows show the difference between the knockdown compared to the control sample.


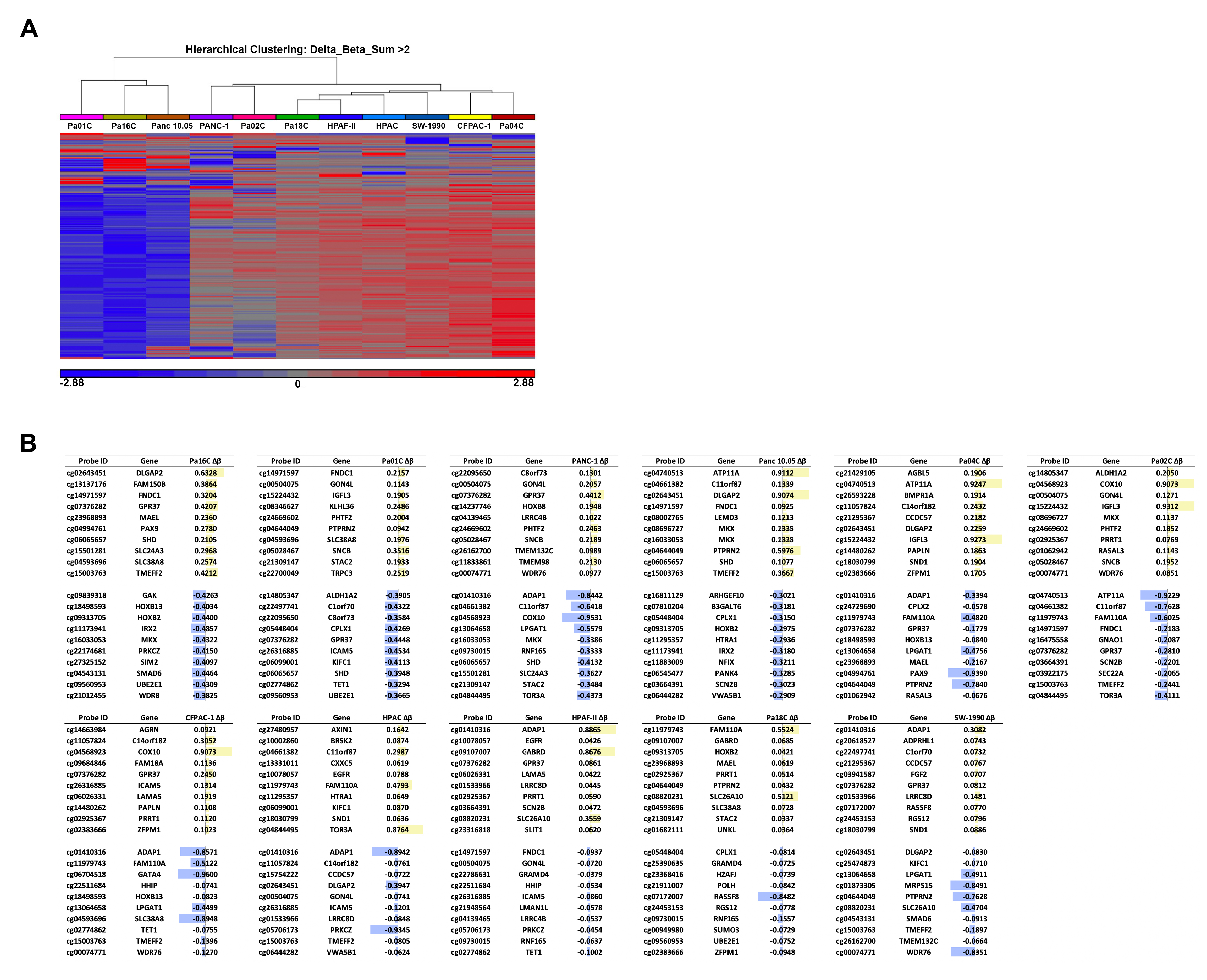


**Supplementary Figure S4. Cell line clustering shows cell-specific differential DNA methylation with KRAS knockdown in pancreatic cells.** **A.** Total of 204 probes used for clustering had Δβ values ≥0.2 or ≤-0.2 in three or more cell line pairs. **B.** Genes corresponding to top 10 differentially methylated CpGs with Δβ values ≥0.2 or ≤-0.2 in at least 3 out of 11 cell line pairs.


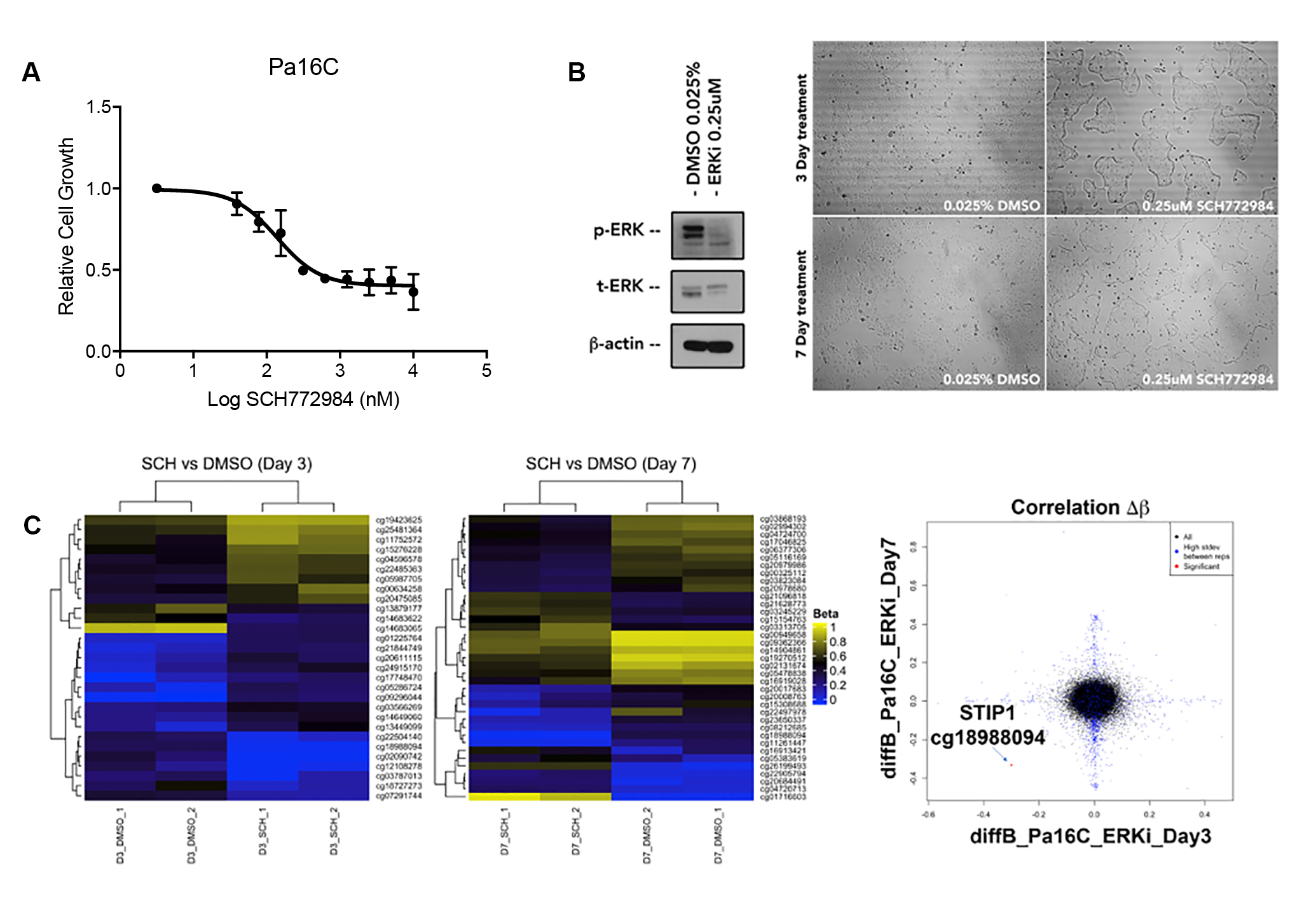


**Supplementary Figure S5. Inhibitor treatment shows limited role for ERK in differential CpG methylation of Pa16C pancreatic cancer cells. A.** Dose-response curve of ERK inhibitior SCH772984 on Pa16C cells. Cell growth was measured by MTT assay 3 days post-treatment. **B.** (Left) Western blot showing relative abundance of total and phosphorylated ERK in Pa16C cells treated with SCH772984 every 48 hours for 7 days. (Right) Phase contrast image of Pa16C cells after 3 and 7 days of treatment. **C.** (Left) Heatmap of differentially methylated probes after 3 days (29 probes) and 7 days (37 probes) of ERK inhibitor treatment based on p-values < 0.05 and Δβ values ≥0.2 or ≤-0.2. (Right) Plot of Δβ values of all probes after 3-day vs 7-day ERKi treatment. Only 1 probe changed in both samples - cg18988094 is hypomethylated in both 3 day and 7 day ERKi treated samples.


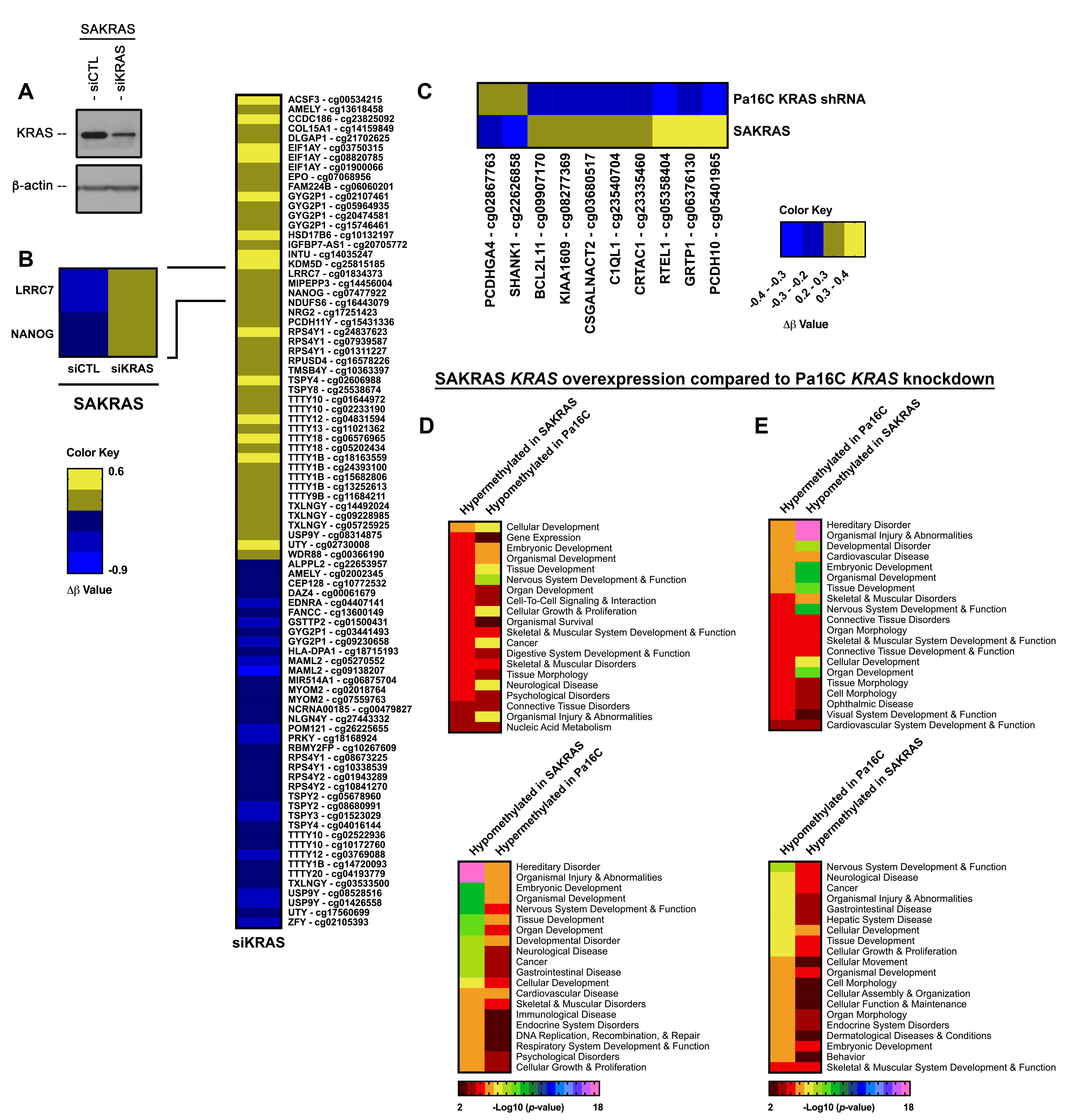


**Supplementary Figure S6. Gene ontology analysis of DM CpGs reveals enrichment of genes involved in development and differentiation associated with changes in KRAS expression.** **A.** Western blot showing KRAS protein expression in SAKRAS cells transfected with siRNA targeting KRAS. **B.** Heat map showing Δβ values of probes with differential methylation in SAKRAS cell transfected with siRNA targeting KRAS. Δβ values in column labeled siCTL are relative to SALEB control cells. **C.** Differentially methylated CpGs that changed in opposite directions in SAKRAS vs SALEB cells compared to Pa16C KRAS shRNA vs Pa16C NS shRNA. **D-E.** Gene ontology analysis of gene promoters that are hypermethylated and hypomethylated in SAKRAS or Pa16C. Heat map of the top 20 overlapping biological processes, sorted by -log10 scale of p-values, which includes a large number of biological processes involved in cell development and differentiation.
