# Supplementary figures and images for: Genome-wide DNA methylation analysis of KRAS mutant cell lines
Source: Sci Rep. 2020 Jun 23;10:10149. doi: 10.1038/s41598-020-66797-x (PMC7311523; doi:10.1038/s41598-020-66797-x)

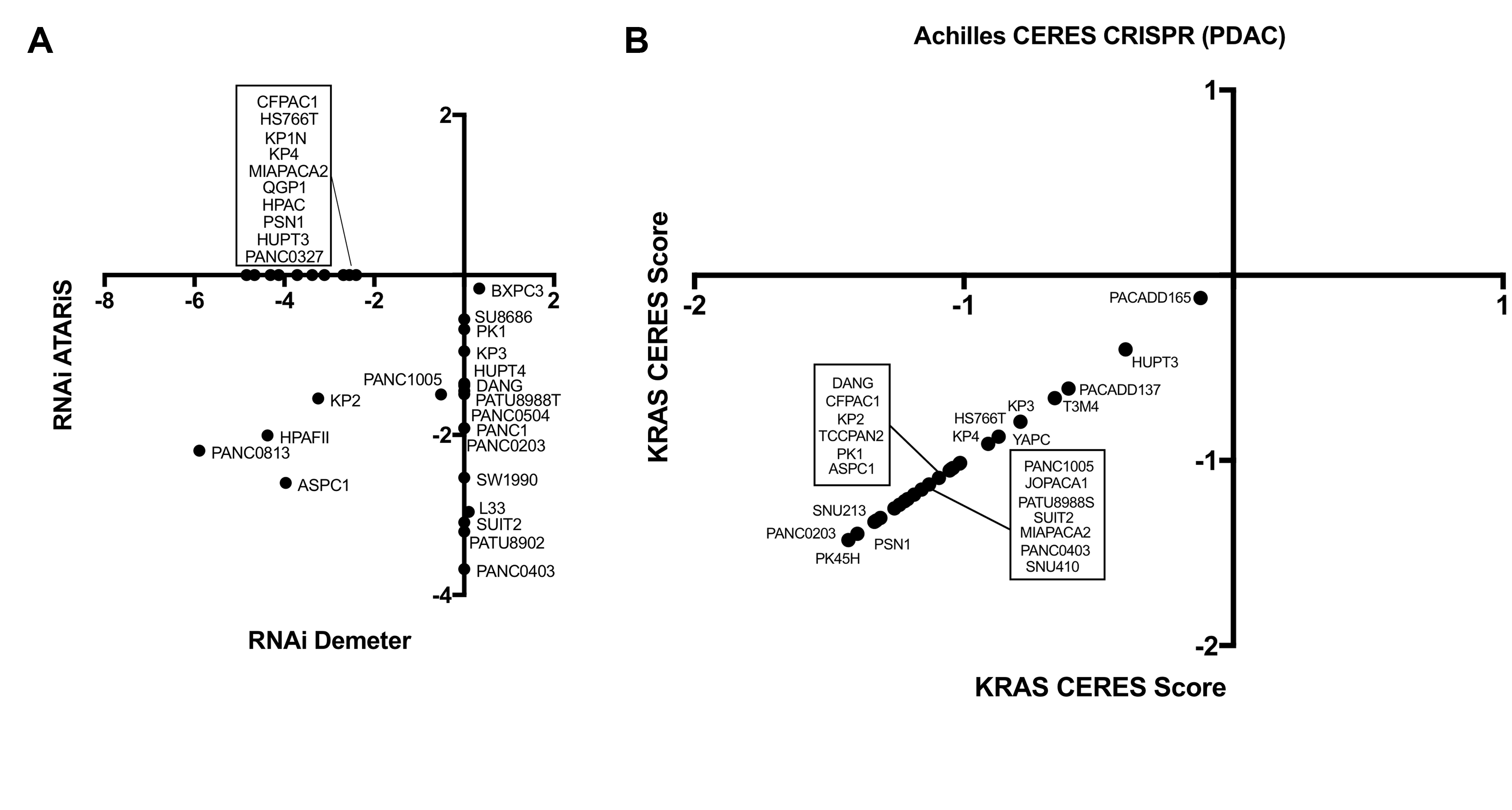

Supplement: Supplementary file 2 — Supplementary Figure S1 [file 41598_2020_66797_MOESM2_ESM.tif]

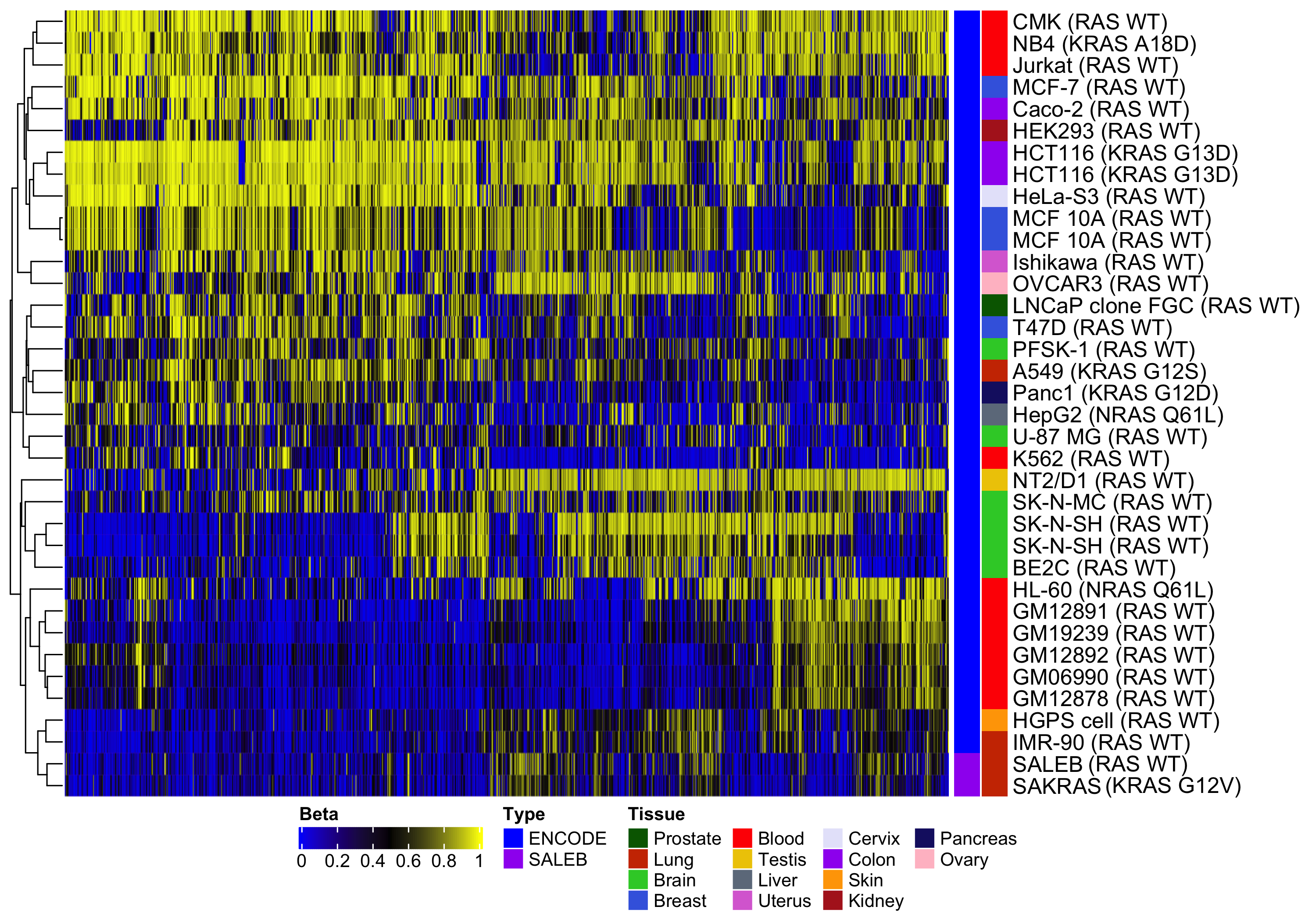

Supplement: Supplementary file 3 — Supplementary Figure S2 [file 41598_2020_66797_MOESM3_ESM.tif]

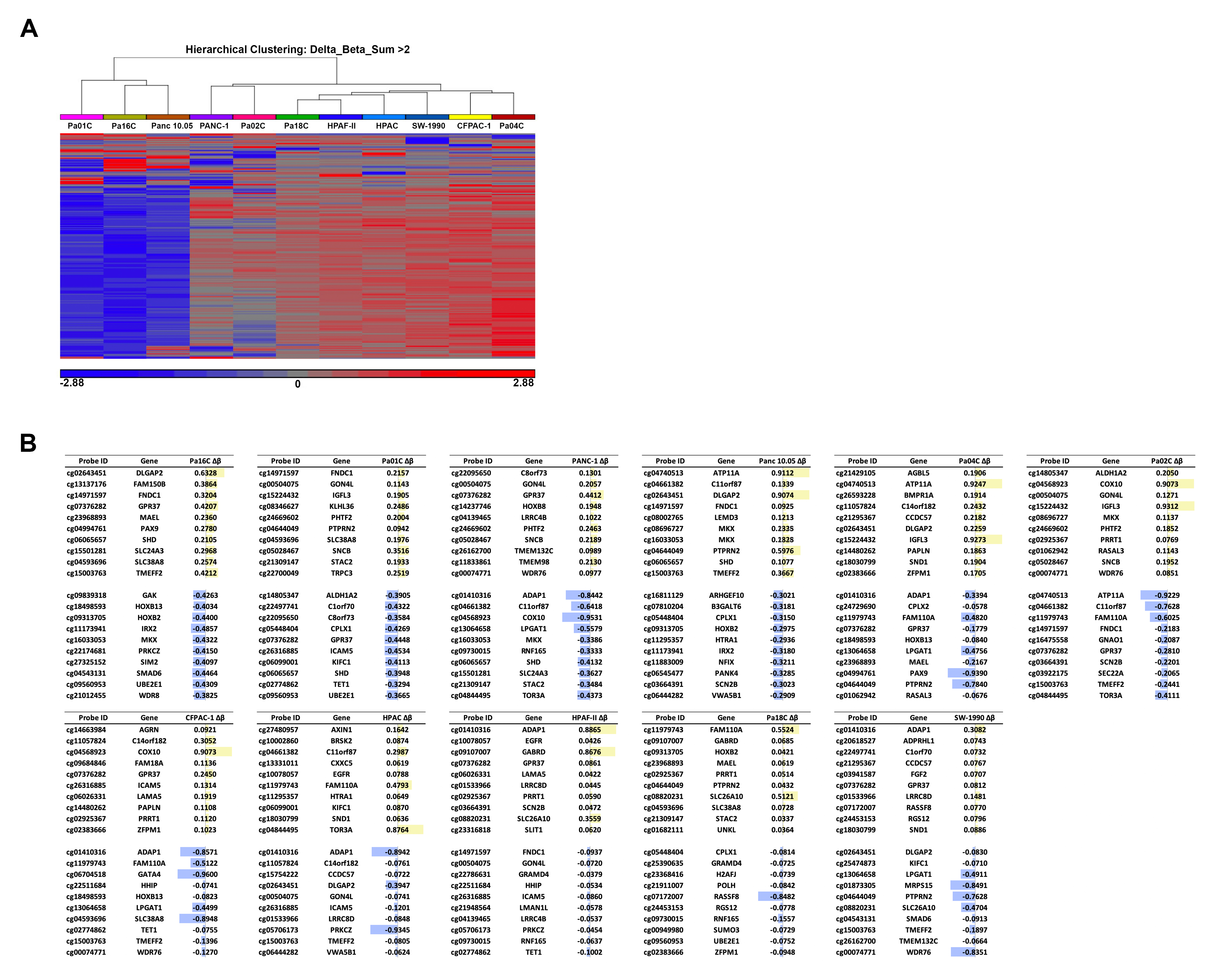

Supplement: Supplementary file 5 — Supplementary Figure S4 [file 41598_2020_66797_MOESM5_ESM.tif]

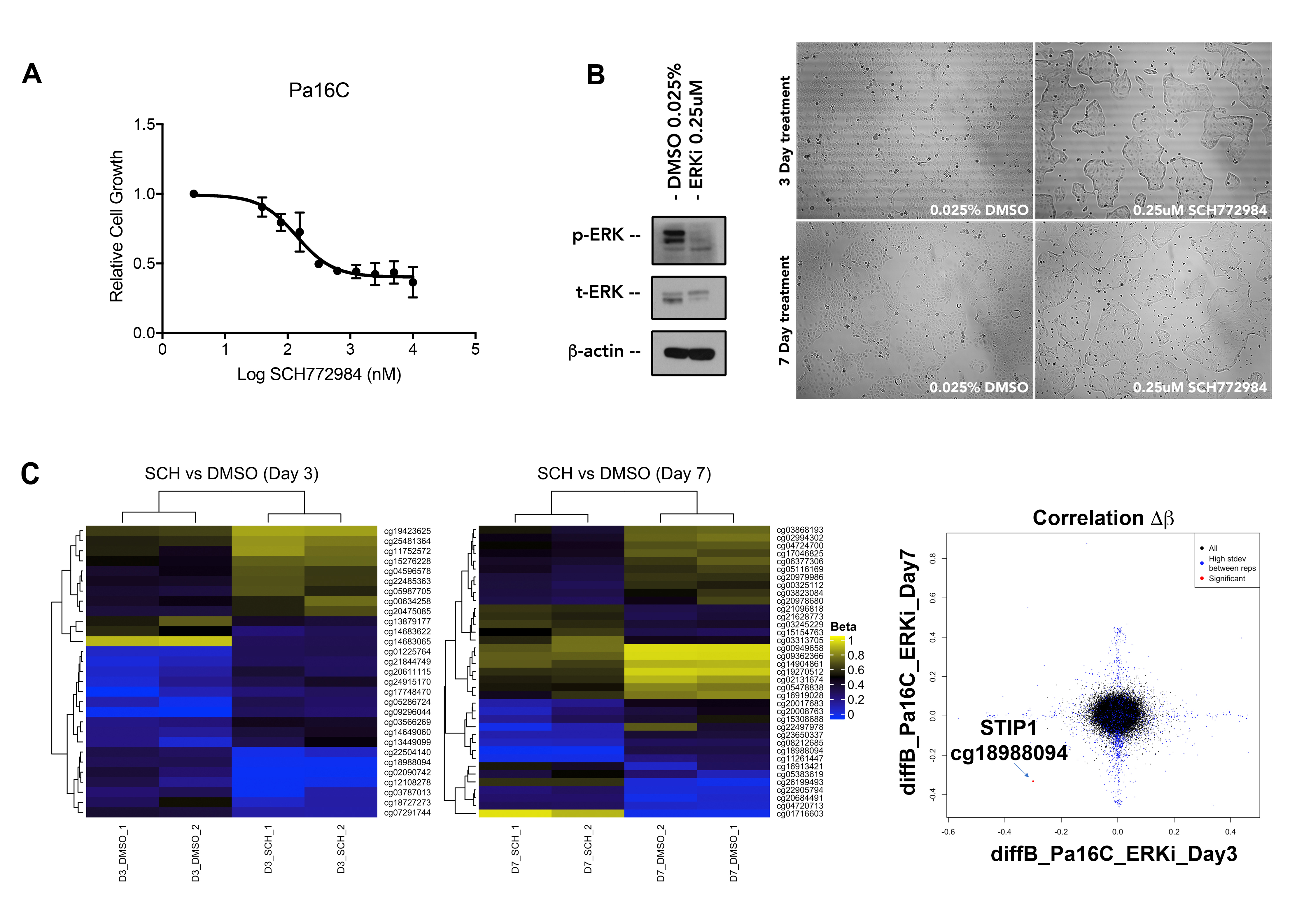

Supplement: Supplementary file 6 — Supplementary Figure S5 [file 41598_2020_66797_MOESM6_ESM.tif]

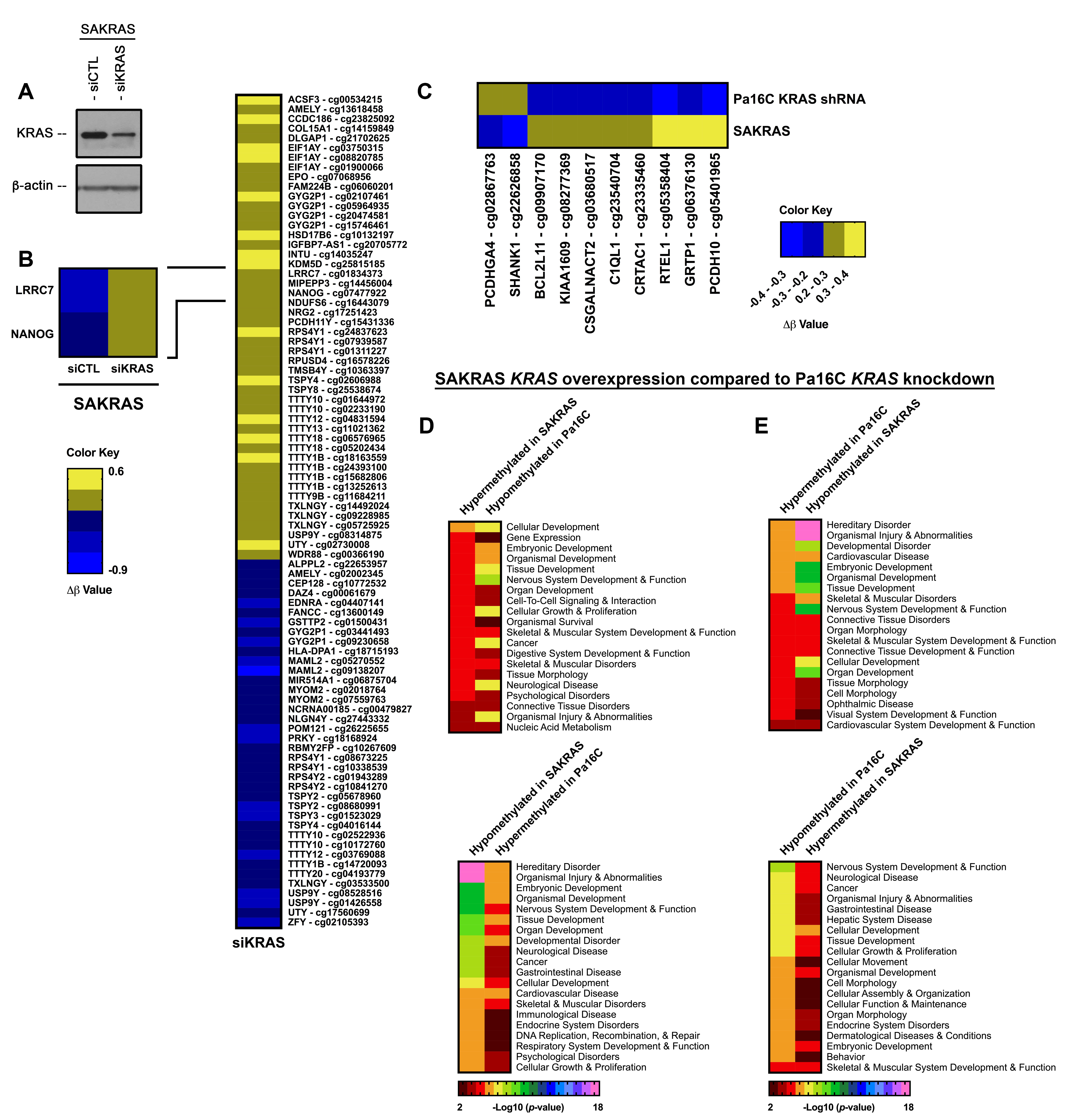

Supplement: Supplementary file 7 — Supplementary Figure S6 [file 41598_2020_66797_MOESM7_ESM.tif]
